# Electronic Health Record–Based Screening for Intimate Partner Violence: A Cluster Randomized Clinical Trial

**DOI:** 10.1001/jamanetworkopen.2024.25070

**Published:** 2024-08-01

**Authors:** Leslie Lenert, Alyssa A. Rheingold, Kit N. Simpson, Dmitry Scherbakov, Michael Aiken, Christine Hahn, Jenna L. McCauley, Naomi Ennis, Vanessa A. Diaz

**Affiliations:** 1 Biomedical Informatics Center, Medical University of South Carolina, Charleston; 2 Department of Psychiatry and Behavioral Sciences, Medical University of South Carolina, Charleston; 3 Department of Healthcare Leadership and Management, Medical University of South Carolina, Charleston; 4 Department of Family Medicine, Medical University of South Carolina, Charleston

## Abstract

**Importance:**

Intimate partner violence (IPV) is a significant public health issue, with a 25% lifetime prevalence. Screening for IPV in primary care is a recommended practice whose effectiveness is debated.

**Objective:**

To assess the effect of an electronic health record (EHR)–based multifactorial intervention screening on the detection of IPV risk in primary care practice.

**Design, Setting, and Participants:**

This cluster randomized clinical trial used a stepped-wedge design to assign 15 family medicine primary care clinics in the Medical University of South Carolina Health System in the Charleston region to 3 matched blocks from October 6, 2020, to March 31, 2023. All women aged 18 to 49 years who were seen in these clinics participated in this study.

**Intervention:**

A noninterruptive EHR alert combined with confidential screening by computer questionnaire using the EHR platform followed by risk assessment and a decision support template.

**Main Outcomes and Measures:**

The main outcomes were the rate at which patients were screened for IPV across the clinics and the rate at which patients at risk for IPV were detected by screening procedures.

**Results:**

The study clinics cared for 8895 unique patients (mean [SD] age, 34.6 [8.7] years; 1270 [14.3%] with Medicaid or Medicare and 7625 [85.7%] with private, military, or other insurance) over the study period eligible for the screening intervention. The intervention had significant effects on the overall rate of screening for IPV, increasing the rate of screening from 45.2% (10 268 of 22 730 patient visits) to 65.3% (22 303 of 34 157 patient visits) when the noninterruptive alert was active (relative risk, 1.46 [95% CI, 1.44-1.49]; *P* < .001). The confidential screening process was more effective than baseline nurse-led oral screening at identifying patients reporting past-year IPV (130 of 8895 patients [1.5%] vs 9 of 17 433 patients [0.1%]).

**Conclusions and Relevance:**

The intervention was largely effective in increasing screening adherence and the positive detection rate of IPV in primary care. A highly private approach to screening for IPV in primary care may be necessary to achieve adequate detection rates while addressing potential safety issues of patients experiencing IPV.

**Trial Registration:**

ClinicalTrials.gov Identifier: NCT06284148

## Key Points

**Question:**

How can screening for intimate partner violence (IPV) be performed efficiently during clinical encounters?

**Findings:**

In this stepped-wedge cluster randomized clinical trial, a high-privacy screening method, which uses the examination computer and noninterruptive clinician alert, was shown to be highly efficient compared with baseline oral screening. The screening rate increased from 45% to 65%, and the new screening tool detected 1.5% of patients reporting past-year IPV, compared with 0.1% of patients detected using the baseline screener.

**Meaning:**

This study suggests that the combination of privacy-preserving procedures and noninterruptive alerting allowed for increased screening rates and sensitivity when screening for IPV.

## Teaser

This cluster randomized clinical trial assesses the effect of an electronic health record (EHR)–based multifactorial intervention screening compared with the standard verbal screening on the detection of risk of intimate partner violence in primary care practice.

## Introduction

Intimate partner violence (IPV) is a significant public health problem, with a 5.9% annual rate of IPV reporting among women in the US,^1^ for which screening in primary care is recommended by the US Preventive Service Task Force.^2^ The lifetime prevalence of IPV is reported to be 25% of all women.^3^

Screening for IPV in primary care is conducted much less frequently than screening for health conditions, such as depression.^4^ Intimate partner violence is stigmatizing to those who are subject to it, and concerns about confidentiality are a recognized barrier to disclosure.^5^ Self-administered computer questionnaires are an effective but underused privacy-preserving approach to screening for IPV^6^; alternatively, many health care professionals often screen for IPV and other issues with oral questions. Regarding IPV, asking questions in an overly routine or uncaring way is a barrier to a forthright response.^7^ Many women, even in emergency department settings with evidence of injuries, choose not to disclose abuse because of privacy and safety concerns.^8^ A lack of expertise by primary care professionals in assessing risk, time constraints, and difficulty linking patients with needed resources may also pose significant barriers for clinicians in the screening processes.^5^

One of the most widely used approaches to change clinician behavior is an alert or prompt in an electronic health record (EHR) for a specific task. Popup-style alerts that block the screen content are the most common type of decision support alert in EHR systems; however, this approach has been overused, and as a result, the alerts are frequently ignored.^9^ An alternative is the use of a noninterruptive alert, which has shown to be effective but has not been studied for IPV screening, to our knowledge.^9^ Another option is a checklist in the EHR that was shown to be effective in the IRIS (Identification and Referral to Improve Safety) trial.^10^

The present study includes implementation of an IPV screening workflow consisting of 4 major components: (1) a noninterruptive alert for annual IPV screening; (2) partner violence screening (PVS) confidential self-report (later also referred to as *high-privacy screener*); (3) patient risk screening with the Danger Assessment-5 (DA-5) instrument^11^; and (4) clinician confidential documentation of screening results, brief intervention, and patient referral.

Components 1, 2, and 3 are described in this article. We compared their effectiveness with an existing nurse-led oral screening checklist, which includes questions related to IPV. We wanted to validate 2 primary hypotheses: that PVS would be more effective than existing nurse-led screening and that the noninterruptive alert would increase the use of both PVS and nurse-led screening.

## Methods

This randomized clinical trial was conducted from October 6, 2020, to March 31, 2023. The Medical University of South Carolina (MUSC) institutional review board waived review because it determined the study was a quality improvement effort that did not require patient consent because identifying and managing IPV was part of its current health care practice and the study addressed how best to operationalize that practice; in addition, the intervention used the Epic software data field protection approaches that decreased patient risks of disclosure. The registration was considered optional by the MUSC institutional review board as the study was deemed quality improvement over the baseline nurse-led screening method already in place. For this reason, the registration was performed post hoc after the trial ended. The trial protocol with the original statistical plan is provided in Supplement 1. This publication was created according to the Consolidated Standards of Reporting Trials (CONSORT) reporting guideline for reporting stepped-wedge cluster randomized trials.^12^

### Context and Intervention

The EHR workflow was designed in the Epic system to present an in-menu, noninterruptive alert that notifies medical assistants to screen annually for IPV each woman between the ages of 18 and 49 years visiting one of the participating clinics. We focused on women of child-bearing age following recommendations of the US Preventive Services Task Force.^13^

Responding to the noninterruptive alert converts the examination room computer to a kiosk-like mode for use by a patient to self-administer a PVS questionnaire. The medical assistant ensures that the patient is alone by removing other adult-age family members from the room so the patient can respond to a 3-item initial screening questionnaire^14^ to detect past-year IPV, which, if positive, cascades to an additional questionnaire administered to assess risk levels of future harm.^11^ The approach provides the maximum feasible level of protection for the privacy of the patient’s responses. If the patient screens positive for IPV, a popup alert notifies the clinician.

The noninterruptive reminder for medical assistants to screen patients remains active until a patient is screened. For patients who screen negative, the noninterruptive reminder for medical assistants is turned off for 1 year. For patients who screen positive, the reminder for medical assistants to screen remains active to encourage repeated screening.

### Control Condition

Our intervention ran parallel to the existing nurse-led oral screening related to IPV, which was used as both a baseline and a control condition. During each visit in which our screening method was assigned to a patient, nurse-led screening was also assigned. This screening method required nurses or medical assistants to complete a checklist in which they indicated, based on professional judgment (eg, they did not directly ask standardized questions), a variety of risk factors. Selecting “caregiver degrades or threatens patient,” “abuse/neglect suspected,” “evaluation for abuse,” “excessive fear/withdrawn or guarded behavior,” or “has unexplained injuries or bruises” was considered a positive indicator for intimate partner abuse risk in the context of the present study. This nurse-led screening was in place before the intervention and was continued throughout the study period.

### Study Design

This study uses a randomized stepped-wedge design,^15^ which was chosen to get adequate statistical power and for pragmatic considerations (sequential activation of the intervention allowed for more efficient training of staff). Clinics were assigned by the research team to 3 blocks matched in size in terms of the number of patients and full-time employees in clinics (Figure). Blocks were activated in a randomized order (using a random number generator).

**Figure.**
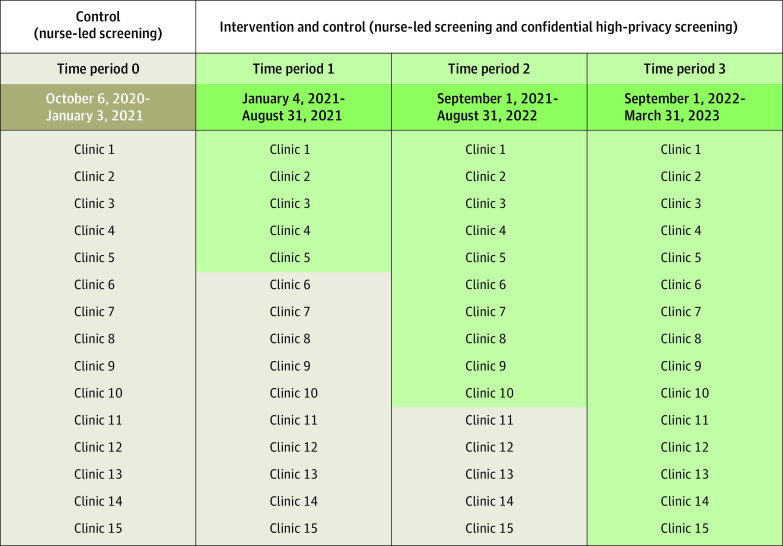
Randomized Stepped-Wedge Design With Clinics Identified Green color indicates the active intervention phase.

To prepare clinics for participation, we worked with the clinical leaders and staff to implement the study. During the initial week of the rollout, Epic research support personnel were available in the clinics to help clinicians learn to use software modifications. Our research team managed the intervention and ensured that it was rolled out in a time-appropriate way. All EHR configurations were tested before according to a project schedule.

### Setting and Sample Size

This study was initially designed for 28 clinics but was conducted in a subset of the 15 larger family medicine–led primary care clinics in the MUSC Health System in the Charleston, South Carolina, region. Family medicine clinics were chosen because their demographics matched our population of interest (as opposed to internal medicine clinics, which serviced an older population). Larger clinics were also selected to address limitations in staff available to support the intervention process.

The study was initially estimated to have 97% power to detect an increase in screening from 0.1% to 5.5% (where 0.1% is IPV prevalence based on *International Statistical Classification of Diseases and Related Health Problems, Tenth Revision* diagnoses according to our preliminary data review in the EHR and 5.5% is the annual IPV prevalence). These calculations were based on 600 patient visits over 6 months per clinic, 8 to 12 clinics per wedge, and 3 wedges, resulting in a total of 34 000 visits in the control and intervention stages. Initial calculations assumed an intraclass correlation coefficient of 0.05 (to account for a moderate level of clustering of patients within clinics), along with an α of .05 and 2-sided testing.

The study required adjustments in response to the COVID-19 pandemic. These adjustments resulted in reductions in the number of clinics and blocks in the randomized design and a shortening of the control run-in period.

### Data Sources and Collection

All data were collected from October 6, 2020, to March 31, 2023, within the Epic EHR system. Data were extracted from Clarity Tables and Chronicles by an experienced full-time research data analyst who acted as an honest broker, anonymizing files before analysis while maintaining patient record linkages. Race and ethnicity data reflect the information collected in the EHR; race and ethnicity were included in analyses to look for effects of these variables on screening rates. This information was used along with other fields from the EHR, such as insurance type and marital status, in statistical models.

### Outcomes

The first primary outcome measure for the study was the rate at which patients were screened for IPV across the clinics. This outcome was calculated as the proportion of patients among all eligible patients who completed IPV questions. We calculated this outcome for both types of screenings (confidential and nurse led) and conducted a separate regression analysis (generalized linear mixed model with a log-link function) to estimate the relative risk (RR) of completing any screening based on demographic variables and based on whether the noninterruptive screening alert was active. This analysis was performed using patient visit as a unit of data, as each unique patient could make several visits to a clinic.

The second primary outcome was the rate at which patients at risk for IPV were detected by screening procedures. The patient was flagged with a positive screening result depending on how they answered the PVS screener questions or how the nurse completed the nurse-led checklist, as described in Table 1. We calculated the proportion of IPV-positive patients detected by each of the screening types, and we conducted a separate regression analysis (generalized linear mixed model with a log link) to estimate how demographic variables and visit type affected the probability of a positive screening result. Responses to individual PVS questions are presented in the form of frequency tables. This analysis was performed using unique patient counts.

**Table 1.** How IPV-Positive Patients Are Identified Using Both Screening Types

Screening type	IPV positive	IPV negative
Nurse-led screening	One or more of the following answers is selected by the nurse during assessment for abuse or neglect: Has unexplained injuries, bruises, cuts Caregiver degrades or threatens patient Abuse or neglect suspected Being evaluated for suspected abuse	One or more of the following answers is selected by the nurse during assessment for abuse or neglect: No suspicion of abuse or neglect Poor hygiene, dirty or unkempt inappropriate clothing Excessive fear, withdrawn, or guarded behavior Noncompliance with plan of care
High-privacy screening	One or more of the following answers was given: Have you been hit, kicked, punched, pushed, shoved, or otherwise hurt by someone at home in the past year? Yes Do you feel safe in your current relationship? No Is there a partner from a previous relationship who is making you feel unsafe now? Yes	The following answers were given: Have you been hit, kicked, punched, pushed, shoved, or otherwise hurt by someone at home in the past year? No Do you feel safe in your current relationship? Yes, or not applicable. Is there a partner from a previous relationship who is making you feel unsafe now? No

Abbreviation: IPV, intimate partner violence.

Secondary outcomes included the severity of risk measured using the DA-5 in IPV-positive cases.^11^ The DA-5 assesses the level of risk for future severe physical injury or possible IPV-related death, with results ranging from 0 (minimal risk) to 5 (maximum risk).

In addition, we collected the acceptability of the screening using a structured survey; patients with positive screening results were asked at the end of the screener if they would be interested in a follow-up interview using the contact method they preferred (email, telephone, or text message).

### Harms

The main risk that we identified was the risk of disclosure to a violent partner. However, our staff made sure that at least a portion of the visit took place when the patient was alone by providing notices in advance to the partner or others accompanying the patient that this is required for medical purposes. Harm was determined by the review of clinician notes in response to the IPV-positive screening.

### Statistical Analysis

Statistical analysis for all outcomes was performed using R, version 4.3.1 (R Project for Statistical Computing) and the MASS package for mixed effects models. All *P* values were from 2-sided tests, and results were deemed statistically significant at *P* < .05.

## Results

During the study period, 17 433 patients (mean [SD] age, 34.1 [8.6] years; 47 American Indian or Alaska Native patients [0.3%], 411 Asian patients [2.4%], 4807 Black or African American patients [27.6%], 26 Native Hawaiian or Other Pacific Islander patients [0.1%], and 11 382 White patients [65.3%]; 2542 [14.6%] with Medicaid or Medicare and 14 891 [85.4%] with private, military, or other insurance) were assigned to nurse-led screening only, and 8895 (mean [SD] age, 34.6 [8.7] years; 17 American Indian or Alaska Native patients [0.2%], 181 Asian patients [2.0%], 2549 Black or African American patients [28.7%], 18 Native Hawaiian or Other Pacific Islander patients [0.2%], and 5732 White patients [64.4%]; 1270 [14.3%] with Medicaid or Medicare and 7625 [85.7%] with private, military, or other insurance) were assigned to both the PVS and nurse-led screening. The demographic characteristics of these 2 groups are provided in Table 2. These numbers correspond to a total of 56 887 patient visits eligible for nurse-led screening and 34 157 visits eligible for PVS and nurse-led screening. The flow diagram describing the flow of participating clinics and patient visits during each time period is provided in the eFigure in Supplement 2. No reports of concern for safety or privacy issues related to the screening procedures were noted in the clinician documentation.

**Table 2.** Demographic Characteristics of the Study Population

Characteristic	Patients, No (%)	
	Group 1: assigned to nurse-led screening (n = 17 433)	Group 2: assigned to confidential screening in addition to nurse-led screening (n = 8895)
Age, mean (SD), y	34.1 (8.6)	34.6 (8.7)
Age group, y		
18-29	5546 (31.8)	2795 (31.4)
30-39	6192 (35.5)	3132 (35.2)
40-49	5695 (32.7)	2968 (33.4)
Race		
American Indian or Alaska Native	47 (0.3)	17 (0.2)
Asian	411 (2.4)	181 (2.0)
Black or African American	4807 (27.6)	2549 (28.7)
Native Hawaiian or Other Pacific Islander	26 (0.1)	18 (0.2)
White	11 382 (65.3)	5732 (64.4)
Other	627 (3.6)	319 (3.6)
Patient refused to answer	79 (0.5)	42 (0.5)
Unknown	48 (0.3)	33 (0.4)
Missing	6 (0.03)	4 (0.04)
Ethnicity		
Hispanic or Latino	738 (4.2)	377 (4.2)
Not Hispanic or Latino	16 404 (94.1)	8353 (93.9)
Refused or unknown	291 (1.7)	165 (1.9)
Insurance		
Private, military, or other	14 891 (85.4)	7625 (85.7)
Medicaid or Medicare	2542 (14.6)	1270 (14.3)
Visit type		
New patient	5639 (32.3)	3126 (35.1)
Other	11 794 (67.7)	5769 (64.9)
Marital status		
Married	7197 (41.3)	3811 (42.8)
Significant other	365 (2.1)	170 (1.9)
Single	8713 (50.0)	4346 (48.9)
Widowed	86 (0.5)	47 (0.5)
Divorced or legally separated	865 (5.0)	421 (4.7)
Unknown	207 (1.2)	100 (1.1)

### Principal Findings

Across the entire study, the PVS questionnaire was completed during 9707 of 34 157 visits in which the high-privacy screening was assigned (28.4%). The triggering of a noninterruptive screening alert had important effects on the overall rate of screening for IPV, increasing the rate of screening using either method from 45.2% (10 268 of 22 730 visits) to 65.3% (22 303 of 34 157 visits) (RR vs without the noninterruptive alert, 1.46 [95% CI, 1.44-1.49]; *P* < .001).

The high-privacy screening was much more effective than the nurse-led screening in identifying patients with potential IPV risk. Only 9 of 17 433 patients (0.1% [95% CI, 0.02%-0.1%]) were identified as being at risk for IPV using the baseline nurse-led screener, while 130 of 8895 patients (1.5% [95% CI, 1.2%-1.7%]) reported past-year IPV with the PVS questionnaire.

### Additional Findings

Table 3 displays the RR of completing any screening type using mixed-effects multilevel analysis. Patients were less likely to complete screening in visits other than their first visit to the clinician (RR, 0.89 [95% CI, 0.88-0.90]; *P* < .001). Patients whose race was other than White were slightly less likely to complete the screening (RR, 0.98 [95% CI, 0.97-1.00]; *P* = .02). Patients who were not married were less likely to complete the screening except for those with unknown marital status (RR, 1.11 [95% CI, 1.07-1.14]; *P* < .001), although only the widowed group and the divorced group reached statistical significance in multilevel analysis (widowed group: RR, 0.91 [95% CI, 0.84-1.00]; *P* = .04; divorced group: RR, 0.93 [95% CI, 0.90-0.96]; *P* < .001). Persons with Medicaid or Medicare were less likely to complete the screening than those with private, military, or other insurance (RR, 0.90 [95% CI, 0.88-0.92]; *P* < .001). The RR of completing any screening was lower for older patients (aged 30-39 years: RR, 0.98 [95% CI, 0.97-1.00]; *P* = .02; aged 40-49 years: RR, 0.98 [95% CI, 0.96-1.00]; *P* = .02). The statistics of answers to individual PVS screener questions are provided in Table 4.

**Table 3.** Results of Multilevel Mixed-Effects Analysis With IPV Screening Completion as the Dependent Variable

Characteristic	Patients, No (%)		Relative risk (95% CI)	
	No screening	Any screening completed	Univariable	Multivariable logistic regression model with clinic as a random effect
Noninterruptive decision support alert				
Not triggered	12 462 (54.8)	10 268 (45.2)	1 [Reference]	1 [Reference]
Triggered	11 854 (34.7)	22 303 (65.3)	1.44 (1.42-1.47)	1.46 (1.44-1.49)
Visit type				
New patient visit	5052 (35.5)	9197 (64.5)	1 [Reference]	1 [Reference]
Other	19 264 (45.2)	23 374 (54.8)	0.85 (0.84-0.86)	0.89 (0.88-0.90)
Race and ethnicity				
White	14 145 (41.4)	20 019 (58.6)	1 [Reference]	1 [Reference]
Other	10 171 (44.8)	12 552 (55.2)	0.94 (0.93-0.96)	0.98 (0.97-1.00)
Marital status				
Married	9227 (41.3)	13 104 (58.7)	1 [Reference]	1 [Reference]
Significant other	506 (43.7)	651 (56.3)	0.96 (0.91-1.01)	0.97 (0.93-1.02)
Single	12 632 (43.3)	16 570 (56.7)	0.97 (0.95-0.98)	1.00 (0.98-1.01)
Widowed	217 (49.4)	222 (50.6)	0.86 (0.78-0.95)	0.91 (0.84-1.00)
Divorced or legally separated	1562 (48.3)	1670 (51.7)	0.88 (0.85-0.91)	0.93 (0.90-0.96)
Unknown	172 (32.7)	354 (67.3)	1.15 (1.08-1.22)	1.11 (1.07-1.14)
Insurance				
Private, military, or other	19071 (41.3)	27 142 (58.7)	1 [Reference]	1 [Reference]
Medicaid or Medicare	5245 (49.1)	5429 (50.9)	0.87 (0.85-0.88)	0.90 (0.88-0.92)
Age, y				
18-29	6619 (40.3)	9789 (59.7)	1 [Reference]	1 [Reference]
30-39	8833 (43.5)	11 487 (56.5)	0.95 (0.93-0.96)	0.98 (0.97-1.00)
40-49	8864 (44.0)	11 295 (56.0)	0.94 (0.92-0.96)	0.98 (0.96-1.00)

Abbreviation: IPV, intimate partner violence.

**Table 4.** Responses of Patients With 1 or More Positive Responses to the High-Privacy Partner Violence Screening Questions

Screening questions and answers	No. (%) (N = 9707)
“Do you feel safe in your current relationship?”	
No	12 (0.1)
Not applicable or prefer not to answer	1366 (14.1)
Yes	8329 (85.8)
Missing	0
Total	9707 (100.0)
“Have you been hit, kicked, punched, pushed, shoved, or otherwise hurt by someone at home in the past year?”	
No	9563 (98.5)
Prefer not to answer	54 (0.6)
Yes	90 (0.9)
Missing	0
Total	9707 (100.0)
“Is there a partner from a previous relationship who is making you feel unsafe now?”	
No	9576 (98.7)
Prefer not to answer	63 (0.6)
Yes	68 (0.7)
Missing	0
Total	9707 (100.0)

### Effect of Demographic Characteristics on the Identification of Patients at Risk for IPV

The eTable in Supplement 2 depicts the results of statistical analysis estimating the likelihood of a positive screening result on the confidential screening. The analysis shows that patients who were not married were much more likely to screen positive for IPV (“significant other” marital status: RR, 2.91 [95% CI, 1.04-8.13]; *P* = .04; single patients: RR, 2.10 [95% CI, 1.28-3.43]; *P* = .003; and divorced patients: RR, 7.91 [95% CI, 4.47-13.97]; *P* < .001). Women with Medicaid or Medicare were more likely to screen positive for IPV than those with private, military, or other insurance (RR, 1.95 [95% CI, 1.33-2.86]; *P* < .001). Other factors, such as race and ethnicity and age, were statistically insignificant in multilevel analysis; patients whose race was other than White had a slightly higher, although not statistically significant, chance of a positive screening result (RR, 1.07 [95% CI, 0.75-1.54]; *P* = .70), and the RR of a positive screening result decreased with age, although not statistically significantly (aged 30-39 years: RR, 0.73 [95% CI, 0.48-1.10]; *P* = .13; aged 40-49 years: RR, 0.67 [95% CI, 0.42-1.07]; *P* = .10).

Follow-up risk screening with the DA-5 occurred among 111 of 130 patients in 125 visits. Of 111 patients completing screening, 55 were at very high risk of future IPV-related physical harm (≥2 positive answers). Only 34 patients had negative responses to all DA-5 questions.

### Acceptability of Screening

Of the patients with a positive screening result for IPV, 59 indicated that they would be interested in a follow-up contact and provided a mode of contact (email, telephone call, or text message), but only 15 responded to the contact. Twelve of the 15 completed the online survey. All 12 participants reported acceptability of being asked the IPV questions during their visit as well as being asked via self-report on the computer.

## Discussion

This study describes a high-privacy IPV screening strategy in the general population of primary care patients. The intervention in this study had 4 components, and the efficacy of 3 of them was shown to be important and potentially effective in this study. The first component was the use of a noninterruptive alert to remind clinicians to screen for IPV on an annual basis. This alert increased the use of both nurse-led oral screening and high-privacy IPV screening. The second component of the intervention was confidential screening by self-report using an examination computer. This process identified many more patients potentially at risk for IPV while also characterizing the risk level of patients identified. Of 125 patients who completed the DA-5, 77 (61.6%) answered one of the DA-5 questions affirmatively. This finding suggests the confidential PVS screening was specific, finding true cases at risk for IPV; it also suggests that the third component is an efficient instrument to validate PVS screener answers. The fourth component of the intervention was a physician decision support system for risk management, which will be described in a separate publication.

There is ongoing debate on how the screening for IPV should be implemented. Current recommendations range from universal screening to clinically informed “case finding” to limiting screening to specific age groups.^16,17^ Given the significant improvement in IPV detection seen in this study using the high-privacy method, we believe that a privately answered examination computer survey is an approach that should be adopted and improved on.

The low rate of identification of IPV by screening in this study does call into question the effectiveness of a universal screening approach in settings of limited resource availability. The observed rate, even with the high-privacy approach, was lower than we anticipated and lower than reported by several (albeit smaller) studies using variations of computer-based screening.^18^

However, the true prevalence of IPV among women in primary care is hard to estimate, as it varies from population to population and is based on the definition of IPV. Besides physical violence, the rate might include other forms of violence, such as verbal, sexual, or emotional abuse.^19^ Many potential barriers to IPV screening are described in the literature.^20,21,22^ Although our study addresses some of the barriers, such as preserving privacy and using a tool that requires minimal staff training, other barriers exist, such as a lack of time and cultural or personal challenges related to disclosure of domestic violence. If IPV disclosure requires that the patient have significant trust in the clinician, then repeated inquiry over time in the context of a primary care relationship may be helpful. Providing more details of the extent of efforts to protect privacy in our screening approach might allow patients to be more comfortable with disclosures. Intimate partner violence screening may also be an area in which randomization of screening methods at a patient level might be necessary to truly understand the effect of variations in methods on individual-specific barriers.

### Limitations

This study has some limitations. The planned rollout of the intervention for this study was interrupted by the COVID-19 pandemic, limiting the time for a control measurement phase. A voluntary approach to screening also has some limitations. Patients who were older, single, from racial and ethnic minority groups or had public insurance were screened less frequently. This finding was particularly concerning in the case of patients with public insurance, as these patients were much more likely to screen positive for IPV risk. The protocol does require extra effort by clinic staff, and an assessment of its effect on clinic productivity may be warranted.

## Conclusions

Intimate partner violence is a prevalent, underdiagnosed, and undertreated problem in primary care environments. This cluster randomized clinical trial found that a 4-part, EHR-based intervention appeared to be highly effective in both increasing the frequency of screening and the percentage of patients identified as at risk for significant future physical IPV-related injuries. However, further work is needed to increase screening and detection rates, as the overall positive rate is still much lower than reported in the literature. Among patients with a positive screening result, the intervention was well accepted.

## Notes

Supplement 1.
Trial Protocol and Statistical Analysis Plan

Supplement 2. **eFigure.** Flow Diagram of Cluster Allocation **eTable.** Results of Univariate and Multilevel Analysis With High Privacy PVS Screen Positive as Dependent Variable

Supplement 3.
Data Sharing Statement
