# Supplementary material for: Electronic Health Record–Based Screening for Intimate Partner Violence: A Cluster Randomized Clinical Trial
Source: JAMA Netw Open. 2024 Aug 1;7(8):e2425070. doi: 10.1001/jamanetworkopen.2024.25070 (PMC11294960; doi:10.1001/jamanetworkopen.2024.25070)
Supplement: Supplement 2. — eFigure. Flow Diagram of Cluster Allocation eTable. Results of Univariate and Multilevel Analysis With High Privacy PVS Screen Positive as Dependent Variable [file jamanetwopen-e2425070-s002.pdf]

Supplementary Online Content

Lenert L, Rheingold AA, Simpson KN, et al. Electronic health record–based screening for intimate partner violence: a cluster randomized clinical trial. *JAMA Netw Open.* 2024;7(8):e2425070. doi:10.1001/jamanetworkopen.2024.25070

eFigure. Flow Diagram of Cluster Allocation
eTable. Results of Univariate and Multilevel Analysis With High Privacy PVS Screen Positive as Dependent Variable

This supplementary material has been provided by the authors to give readers additional information about their work.

© 2024 Lenert L et al. *JAMA Network Open.*

eFigure. Flow Diagram of Cluster Allocation

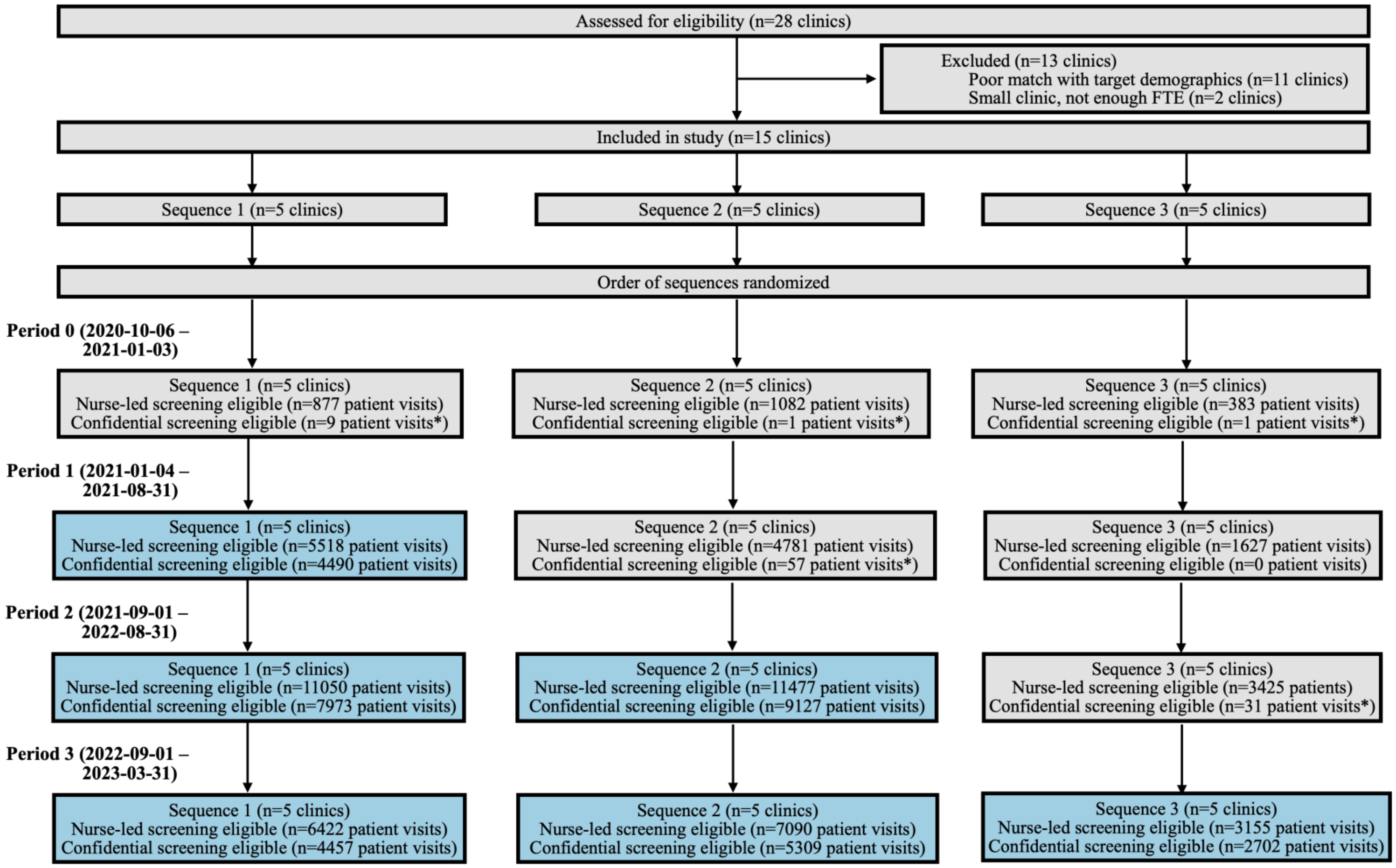

Flow chart of cluster allocation with patient visits during each period displayed. * Denotes test patient visits. Blue shade reflects intervention condition, gray shade – control condition.

© 2024 Lenert L et al. *JAMA Network Open.*

eTable. Results of Univariate and Multilevel Analysis With High Privacy PVS Screen Positive as Dependent Variable

		Not positive	Positive	RR (univariable)	RR (multivariable model with clinic as a random effect)
Visit type	New Patient	3070 (98.2)	56 (1.8)	-	-
	Other	5693 (98.7)	76 (1.3)	0.74 (0.52-1.04, P=0.08)	0.73 (0.52-1.02, P=0.07)
Race	White or Caucasian	5658 (98.7)	74 (1.3)	-	-
	Other	3105 (98.2)	58 (1.8)	1.42 (1.01-2.00, P=0.04)	1.07 (0.75-1.54, P=0.7)
Marital status	Married	3788 (99.4)	23 (0.6)	-	-
	Significant Other	166 (97.6)	4 (2.4)	3.90 (1.36-11.15, P<0.01)	2.91 (1.04-8.13, P=0.04)
	Single	4266 (98.2)	80 (1.8)	3.05 (1.92-4.84, P<0.01)	2.1 (1.28-3.43, P<0.01)
	Widowed	46 (97.9)	1 (2.1)	3.53 (0.49-25.57, P=0.19)	3.14 (0.46-21.56, P=0.24)
	Divorced/Legally Separated	399 (94.8)	22 (5.2)	8.66 (4.87-15.40, P<0.01)	7.91 (4.47-13.97, P<0.01)
	Unknown	98 (98.0)	2 (2.0)	3.31 (0.79-13.86, P=0.08)	2.28 (0.56-9.32, P=0.25)
Insurance	Private/Military/Other	7533 (98.8)	92 (1.2)	-	-
	Medicaid/Medicare	1230 (96.9)	40 (3.1)	2.61 (1.81-3.77, P<0.01)	1.95 (1.33-2.86, P<0.01)
Ethnicity	Not Hispanic or Latino	8228 (98.5)	125 (1.5)	-	-
	Hispanic or Latino	372 (98.7)	5 (1.3)	0.89 (0.36-2.15, P=0.79)	-
	Refused/Unknown	163 (98.8)	2 (1.2)	0.81 (0.20-3.25, P=0.76)	-
Age	18-29	2739 (98.0)	56 (2.0)	-	-
	30-39	3092 (98.7)	40 (1.3)	0.64 (0.43-0.95, P=0.03)	0.73 (0.48-1.1, P=0.13)
	40-49	2932 (98.8)	36 (1.2)	0.61 (0.40-0.92, P=0.02)	0.67 (0.42-1.07, P=0.1)

© 2024 Lenert L et al. *JAMA Network Open.*
