# Supplementary material for: Electronic Health Record–Based Screening for Intimate Partner Violence: A Cluster Randomized Clinical Trial
Source: JAMA Netw Open. 2024 Aug 1;7(8):e2425070. doi: 10.1001/jamanetworkopen.2024.25070 (PMC11294960; doi:10.1001/jamanetworkopen.2024.25070)
Supplement: Supplement 3. — Data Sharing Statement [file jamanetwopen-e2425070-s003.pdf]

Data Sharing Statement

Lenert. Electronic Health Record–Based Screening for Intimate Partner Violence. *JAMA Netw Open*. Published August 01, 2024. doi:10.1001/jamanetworkopen.2024.25070

Data
Data available: Yes
Data types: Deidentified participant data
How to access data: The data can be accessible by making a request to Dr. Leslie Lenert (Lenert@musc.edu)
When available: With publication

Supporting Documents
Document types: None

Additional Information
Who can access the data: Anyone requesting the data
Types of analyses: For any purpose
Mechanisms of data availability: With a signed data access agreement
