# Supplementary material for: Electronic Health Record–Based Screening for Intimate Partner Violence: A Cluster Randomized Clinical Trial
Source: JAMA Netw Open. 2024 Aug 1;7(8):e2425070. doi: 10.1001/jamanetworkopen.2024.25070 (PMC11294960; doi:10.1001/jamanetworkopen.2024.25070)
Supplement: Supplement 1. — Trial Protocol and Statistical Analysis Plan [file jamanetwopen-e2425070-s001.pdf]

Introduction to the Revised Proposal

We thank the reviewers for their helpful comments. We have reorganized the application and clarified issues raised. Perhaps our most significant addition is data from the use of PRO techniques in MUSC clinics over the past 9-months. These results show our workflow for PRO capture is well accepted by both clinic staff and by patients and eminently feasible to adapt to IPV screening. We have added detail on patient reported outcomes also expanded the human subjects section addressing issues raised by reviewers. We have added material on specific dissemination plans. Other responses to summary comments are below:

"Patient centered design was not addressed clearly"—Human factors experts added to the research team (Dr. Welch and his mentor Dr. Madathil) will review existing processes for PRO capture and optimize those from the patient perspective. An additional important area for human factors work from the patient perspective will be the hand off to the national hotline for initiation of counseling.

"The team has not collaborated in the past"—The entire team has not collaborated in the past but dyads and triads of researchers have collaborated. For example, Dr. Lenert and Dr. Simpson collaborate on an PCORI back pain study described herein. Drs. Rheingold, McCauley, and Moreland have collaborated on various DOJ and SAMHSA funded projects related to victims of crime.

"It is not clear the hotline will be available enough of the time" The National Domestic Violence offers access to trained counselors 24/7. It would be hard to match this with local resources. From a generalizability standpoint, local resources across communities vary in scope and access. Use of the Hotline makes this intervention much easier to disseminate.

"Dissemination plans are not clear"—We have added additional information about dissemination approach. Dissemination plans includes "open sourcing" modifications of Epic and training materials to the Epic community; presentations at Epic user meetings and posting of materials on the Epic User Web; and working with regional partners through Health Sciences South Carolina (which integrates 8 distinct health systems) to accelerate dissemination efforts.

"The plan for the protection of human subjects there was the concern that risks to participants are not explicated"—We have added more details on protection of human subjects. One of the PI's (Rheingold) is the Director of Clinical Operations at the National Crime Victims Research and Treatment Center and is highly experienced in work with at risk populations, especially interpersonal violence victims. Please see the expanded section.

"It's unclear which patients will be human subjects and which will not" and "Given the sensitive nature of the project topic, more details should be provided on informed consent, procedures, risks, and protections against risks in surveying patients about the IPV process and tools." -- Patients whose records from clinical are used in an anonymized fashion are not considered human subjects for purposes of this study. Anonymization is performed by honest brokers affiliated with the MUSC CTSA and every care will be taken to prevent accidental data breaches prior to anonymization. Patients consenting (orally) for follow up interviews are considered human subjects, and every effort will be taken (see Human Subjects section) to protect their safety in follow up interviews. See new details added to the Human Subjects section including a description of the protocol research assistants will follow in obtaining follow up data. Data from these interviews will be anonymized and linked by honest brokers prior to creation of analytic files to further reduce risks.

Specific aims:

This application seeks to demonstrate, at a single institution, the impact of adopting an informatics-based approach to screen, assess and refer patients for counseling for Intimate Partner Violence (IPV) using self-administered patient-reported-outcomes questionnaires and other electronic health records (EHR) tools, creating a platform for rapid dissemination of this system to other sites with the Epic EHR.

Violence against women in the US is a serious public health problem and a human rights issue . The Bureau of Justice Statistics reports that 22% of violent crimes against women are accounted). The US Preventive Services Task Force (USPTF) recommends IPV screening for women of childbearing age, indicating that such screening would have a moderate net public health benefit . However, the overall prevalence of screening for intimate partner violence (IPV) in health care settings is still low (O'Doherty, Taft et al. 2014). Moreover, evidence on the effectiveness of screening programs are conflicting (O'Doherty, Taft et al. 2014). Recent studies indicate that, while screening increases the rate of identification of IPV, it has not been shown to increase rates of referral for IPV counseling or improve health outcomes (O'Doherty, Taft et al. 2014). Evidence also show that effective IPV screening programs are those with a "systems level" approach to design and implementation (Miller, McCaw et al. 2015, Young-Wolff, Kotz et al. 2016). For example, Northern California Kaiser Permanente (NCKP) has implemented one of the most successful and effective programs to create a healthcare system optimized for IPV care (Miller, McCaw et al. 2015) This application describes our efforts to adapt and fully integrate the NCKP program into an EHR in general outpatient care settings. The proposed intervention includes several elements: tools for presenting and capturing patient-entered responses to screening questions, decision support providers for screening and detection of IPV, tools for referral to national IPV counseling resource via telephone/telehealth, and EHR modifications to support safe documentation and billing for IPV services. The following two aims are carefully designed to accomplish all elements of the study designing, implementing and evaluating a health system-level intervention through EHR modifications.

Aim 1: Based on lessons learned from the successful IPV screening program at Northern California Kaiser Permanente, and innovations in questionnaire administration developed at MUSC, create a replicable systematic process for using an electronic health records system (EHR) screening patients for IPV, detecting those at risk and facilitating their referral and entry into counseling. Specifically, the system will: (A) manage tracking and administering an IPV screen questionnaire once a year to a vulnerable population of patients ; (B) alert providers of patient responses that reflect potential IPV; and, (C) assists providers with identification and documentation of IPV risk while maintaining special confidentiality for IPV documentation in the record; (D) assist providers with tracking of patients at risk for IPV and with initiation of referrals for IPV counseling. The software combines proven IPV screening instruments with assessment, documentation and intervention tools for EHRs into a replicable automated system of care.

Aim 2: To deploy and test this automated system for IPV care in MUSC primary care and women's health clinics. We hypothesize that the integration of screening, diagnosis and counseling, referral to support services, and billing tasks within an EHR can lower barriers and increase referral rates.

The implementation (Aim 2) will follow a stepped wedge cluster randomized design at MUSC primary care and women's health clinics to support a high-quality evaluation. To evaluate the effectiveness and the impact of the system, rates of IPV diagnosis and referral of women for counseling services under the newly developed system will be compared to those under usual care for IPV using each clinic as its own control. "Usual care" for IPV at MUSC in primary care includes training and awareness activities around IPV diagnosis and care. The impact of advanced IPV care system on provider productivity and reimbursement rates will also be measured as part of the evaluation plan to assess impacts on sustainability. Other outcome metrics will include patient well-being measures, satisfaction and provider satisfaction with IPV tools. If shown to be effective, it is our intention that the IPV tools created under this effort could become part of the base Epic system distributed by the manufacturer, and hence integrated into the EHRs to care 190 million patients across the US. By the completion of this wok, we will have a developed and validated system for IPV care that is evidence-based and easy to disseminate from one institution to another. Demonstration of potential to scale across multiple sites will be the focus of future work. However, this set of Epic modifications will be made available in an open-source style approach to the Epic community at the earliest possible date.

A. Significance:

A.1. IPV burden in the US:

Violence against women in the US is a serious public health problem and a human rights issue. The Bureau of Justice Statistics reports that 22% of violent crimes against women are accounted for by Intimate Partner Violence (IPV), which is defined as physical violence, sexual violence, stalking, and/or psychological aggression (including coercive acts) by a current or former intimate partner . Up to one in three women and one in four men report being a victim of physical violence by an intimate partner at least once in their lifetime, with 25% of women reporting severe forms of IPV (Breiding 2014). In addition to high rates of death resulting from IPV, one-half of female IPV victims reported being physically injured and 18.5% were treated for their injuries within a healthcare system . IPV victims are more likely than non-victims to report having asthma, diabetes, and irritable bowel syndrome, as well as frequent headaches, chronic pain, difficulty with sleeping, activity limitations, poor physical health, and poor mental health as compared to men and women who have not experienced IPV [6]. Further, IPV victims often develop chronic mental health problems, including depression, posttraumatic stress disorder, anxiety, substance abuse, and high risk for suicide (Campbell 2002, Campbell, Webster et al. 2009, Silverman and Loudon 2010, Moyer 2013). The health-related costs of IPV exceed $5.8 billion annually . Of that amount, nearly $4.1 billion are for direct medical and mental health care services, and nearly $1.8 billion are for the indirect costs of lost productivity or wages. Prevalence of IPV is equal to breast cancer, thyroid problems, and colon cancer suggesting that health care systems should be equipped to effectively screen, identify, and improve access to resources (Walters, Chen et al. 2013).

Many IPV cases go unreported due to stigma, fear, shame, and reprisal (Wingood, DiClemente et al. 2001, Brock 2003). Additionally, less than two in five IPV victims injured during their most recent victimization received any kind of medical treatment (Letourneau, Holmes et al. 1999). Even so, the health care system appears to be a potential setting to improve IPV detection and care. Evidence suggests that individuals are most likely to report IPV to their healthcare provider, and are also more likely to utilize interventions suggested by their healthcare provider compared to any other official personnel (McCloskey, Lichter et al. 2006). Importantly, findings have demonstrated that, at 2- year follow-up, women provided with referrals directly from the healthcare provider reported less violence and assault risk, as well as decreased healthcare costs (McFarlane, Groff et al. 2006). Consequently, screening and counseling for IPV is identified by the Institute of Medicine (IOM) as one of eight key preventive services that would help ensure women’s health and wellbeing (IOM, 2011.)

A.2. Gaps in Evidence:

The US Preventive Services Task Force (USPTF) recommends IPV screening for women of childbearing age, indicating that such screening has a moderate net public health benefit . Following USPTF recommendations, the Health Resources and Services Administration (HRSA) developed guidelines inserted into the Affordable Care Act (ACA) to require routine IPV screening and counseling as a preventive service for adolescent and adult women (Oehme and Stern 2014). Other organizations, including the American Medical Association (AMA), American Congress of Obstetrician Gynecologists (ACOG), and the American Nurses Association (ANA) have recommended screening for IPV across health care specialties, including primary care settings. Screening recommendations support universal screening to include all women and not only those with obvious signs of physical abuse. Consequently, the demand for training for IPV screening among healthcare professionals has increased. Several screening instruments have demonstrated high levels of sensitivity and specificity for identifying IPV in health care settings, including primary care. Further, patient-administered computerized screening has demonstrated patient acceptability and increased rates of IPV discussion, disclosure and service provision (Rhodes, Lauderdale et al. 2002, Nelson, Bougatsos et al. 2012). Despite this progress, the overall prevalence of consistent systematic screening for IPV in primary care settings remains low and the effectiveness of the screening programs on improving ultimate health outcomes is still unclear. A recent meta-analysis (O’Doherty, Taft et al. 2014) revealed a gap in evidence regarding referrals following screening. Specifically, evidence indicates that, *while screening is proven to increase the rate of identification of IPV, it has not been shown to increase rates of referral for IPV counseling.* Even so, there are a number of moderate sized trials that have demonstrated the efficacy for selected outcomes: reducing exposure to IPV, morbidity, and mortality (Nelson, Bougatsos et al. 2012, Kottenstette and Stulberg 2013).

Many of the screening programs included in the meta-analysis by O'Doherty and colleagues lacked a "systems level" approach to implementation, as described by Miller and colleagues (Miller, McCaw et al. 2015, Young-Wolff, Kotz et al. 2016). Using this "systems level" methodology, Northern California Kaiser Permanente(NCKP) has implemented one of the most successful and effective programs to create a healthcare system optimized for IPV care (Young-Wolff, Kotz et al. 2016). The approach is designed to transform the entire organization to focus on IPV detection and care but is difficult to implement. The intervention areas of this program are classified into four components: (1) a supportive environment, (2) links to supportive organizations, (3) systematic inquiry and referral integrated into the electronic health record, and (4) on-site IPV services. The breadth of the intervention at NCKP is remarkable — from posters on the wall, to provider training, major EHR modifications, and creating new avenues for follow up on positive findings. Based on historical, non-randomized data, this approach increased the annual IPV case recognition from about 1000 per year in 2000 to 8300 per year in 2013 in the Northern California region. These results suggest the NCKP model is a potentially *nationally replicable* model (Miller, McCaw et al. 2015, Young-Wolff, Kotz et al. 2016). However, we believe that replication of the full extent of the shift in culture that occurred in NCKP might be difficult, especially if strong support for the required organizational change at a leadership level was lacking. An additional concern for reproducibility, is that the intervention was carried out in the cultural context of a unique vertically integrated managed care organization without requirements for fee-for-service billing and other tools necessary for financial sustainability. In addition, special privacy and security protections are implemented at an organizational level in our proposed implementation to enhance patients' safety.

A.3. The current study

The current study will address whether an NCKP approach, modularized and adapted for dissemination, improves IPV care within a primary care setting by increasing screening, detection, and referral for care. Specifically, we focus on the value of health system level changes implemented through electronic health record (EHR) modifications, including tools and decision support for screening and detection of IPV, assessment by a primary care provider and a referral for counseling with "warm handoff" via telephone to a national IPV counseling resource. Additional EHR modifications will support safe billing for provider IPV services to help ensure financial sustainability of the program. The goal is the creation of an easily disseminated EHR-based program for screening and referral.

This study focuses on integration of screening with diagnostic tools and a scalable treatment system—referral to National IPV Hotline for counseling. The national IPV Hotline provides access to trained counselors 24/7 who can help patients begin the process of learning how to protect themselves from IPV. A national hotline is being used as our counseling resource as often local advocacy organizations and shelters rely on volunteers who may vary in training and approach. By using the National Hotline will also allow for ease of dissemination and expanded implementation. We will systematize the "handoff" from EHR to the hotline using telehealth technologies (removing requirements for exam room telephones) and document successful handoffs in the EHR in provider notes.

This study also explores the impact of IPV screening on patient satisfaction to help ensure that screening improves the overall patient experience in the primary care clinic. NCKP has not published data on the effect of the intervention on patient satisfaction. It also measures effects of IPV screening and counseling on providers time and work flows and on their clinical productivity in resource valuation units (RVUs).

B. Innovation

The proposed research uses randomized stepped-wedge design across multiple clinics in a single healthcare organization that should produce a high-quality evidence on its impact. Future work will examine the intervention dissemination across multiple sites. This design is innovative in the context of evaluation of IPV interventions. This kind of dissemination research is needed because, despite strong recommendations for IPV screening at a national level, evidence for the overall effectiveness of screening on health outcomes is inconsistent and may be a barrier to adoption. Specifically, evidence for the impact of EHR-based IPV screening in primary-care settings on rate of initiation of counseling is an undeveloped area. Understanding of the link between screening and initiation of counseling has been identified as a critical evidence-gap by the

USPTF and US Dept. of Health and Human Services (de Boinville 2013, Moyer 2013). In addition, a recent review of IPV referral programs and processes strongly recommended the further evaluation of the effectiveness of referral processes for IPV in health care settings (Kirst, Zhang et al. 2012).

An additional gap is the lack of data on the economic impact on providers of screening and counseling for IPV. Concerns about negative impacts on productivity may be barrier that prevents some providers and organizations from zealously screening, providing medical counseling and initiating referrals for care. This study includes development of tools for secure, safe billing for IPV, enhancing the sustainability of the intervention. The impact of screening on practice Resource Value Units is measured as a secondary outcome as part of the evaluation plan.

The proposed research focuses on making the NCKP more feasible to disseminate. From a dissemination science perspective, the NCKP model nationally requires its abstraction into a *replicable intervention.* This abstraction is our primary innovation. An effective, well-specified EHR-based screening, diagnosis, and referral protocol, integrated with national hotline is the kind of intervention that could be disseminated to many sites. The work proposed creates a *single, comprehensive IPV care for an EHR system*. If shown to be effective, the IPV tools created under this effort could become part of the base Epic system distributed by the manufacturer, and hence integrated into the EHRs to care 190 million patients across the US.

Our main technical innovations is additions to Epic to support administration of the IPV PRO questionnaires to patients on a recurring basis. This application uses a novel workflow developed at MUSC that tracks what questionnaires a patient needs to complete on a given visit and allows the exam room computer (i.e., the doctor's computer) to be safely used by patients to complete self-administered questionnaires. This allows PRO capture without purchase and maintenance of additional hardware (a tablet may be used optionally if required by clinic layout.) This workflow operates completely within the Epic system—no additional software or web service interaction is required. Questionnaires for IPV screening appear automatically in the workflow on an annual basis. The workflow is well-validated at MUSC, having been used in thousands of patients.

A second technical innovation, is the automatic protection of information for patients who screen positive for IPV in a confidential department in Epic. This segmentation of information prevents inadvertent disclosures of information about IPV to a patients' partner, which could provoke violence to the patient. A novel alerting system helps link the routine care in each clinic with the confidential department. In settings where providers are less focused on IPV care than NCKP, this innovation may increase patients' safety by reducing provider effort to maintain security while making them more cognizant of the need for it through EHR alerts.

C. Approach

C.1. Overall Research design:

This study objective is implementation research focused on the use of informatics and the EHR to improve the care process for IPV victims. We seek to abstract, refine and demonstrate portability of defined components from programs at Northern California Kaiser Permanente (NCKP) for screening and referral of patients experiencing IPV. The focus of this effort is to draw upon NCKP's program to create a set of modular tools that are effective for screening and initiation of IPV care, though perhaps not as comprehensive as the KP full approach. Our intervention is a structured optimization of a widely-used EHR system (Epic) for IPV screening, diagnosis and management, in conjunction with linkage to a scalable intervention for patients at risk for IPV - a national 800 number for IPV counseling and care. An advisory board including internal and external stakeholders will oversee the design, the implementation and the evaluation of the study (see LOS).

C.2. Conceptual model:

C.2.1 *The RE-AIM model*

The formal model that guides the design and evaluation of this study is the RE-AIM model as adapted for health information technology applications (Bakken and Ruland 2009):

REACH describes the range of persons in a healthcare population that would be potentially impacted by the program. The intervention is designed to target the entire female population receiving primary care.

EFFICACY/EFFECTIVENESS is assessed at the individual level. As described below in aim 2 and in the evaluation plan, our intervention focuses on diagnosis of IPV or identification of potential for harm by the clinician and referral to an IPV counseling resource. The rates of both outcomes are studied. Untoward effects of IPV care will also be explored. In patients who are diagnosed with IPV, we will follow those patients in a confidential registry, and the impact of IPV care will be ascertained.

ADOPTION in this setting will be examined on a clinic-by-clinic basis and characterized based on proportion of patients screened between rooming and the provider visit. Training tools and ongoing feedback to clinics on use of the IPV screening system supports adoption.

IMPLEMENTATION in this setting is viewed as the rate of successful completion of screening in general patients, as well as rates of completion of counseling by providers in patients who are identified as high risk, and rates of referral and initiation of counselling by patients. Provider surveys will inform us of barriers to adoption and address clinicians' satisfaction. Patient satisfaction will also be addressed.

MAINTENANCE in this setting refers to the feasibility and costs of ongoing operation of the program after the study. Several key cost factors will be evaluated. Overall visit length and the duration of time required for completion of the questionnaire will be measured using Epic log files. Effects on provider productivity will be measured using resource value units (RVU). Costs of access to IPV counseling via National Domestic Violence Hotline will be tracked.

C.*3. AIM 1: Adapt existing NCKP tools for IPV for automated workflow and rapid dissemination*

Aim 1 focuses on developing an integrated suite of EHR tools that automates "best practices" from NCKP for screening and intervention for IPV identified within clinical settings by *prompted disclosure* (Figure 1). Other sections detail choice of questionnaires for screening tools, diagnostic tools, and other elements. For this study, we plan to use the Partner Violence Screening (PVS) for universal screening; however, we will gather input from our advisory board on final screening tool selection. The PVS is a three-item brief instrument assessing past year IPV and has been used in emergency departments and primary care practices. The instrument demonstrates good sensitivity and specificity as well as established predictive validity. It is similar to the questionnaire used by NCKP but it has been formally validated. (Feldhaus et al., 1997, Houry et al. 2004, Mills et al, 2006) The items are:

- *Have you been hit, kicked, punched, or otherwise hurt by someone within the past year? If so, by whom?*
- *Do you feel safe in your current relationship?*
- *Is there a partner from a previous relationship who is making you feel unsafe now?*

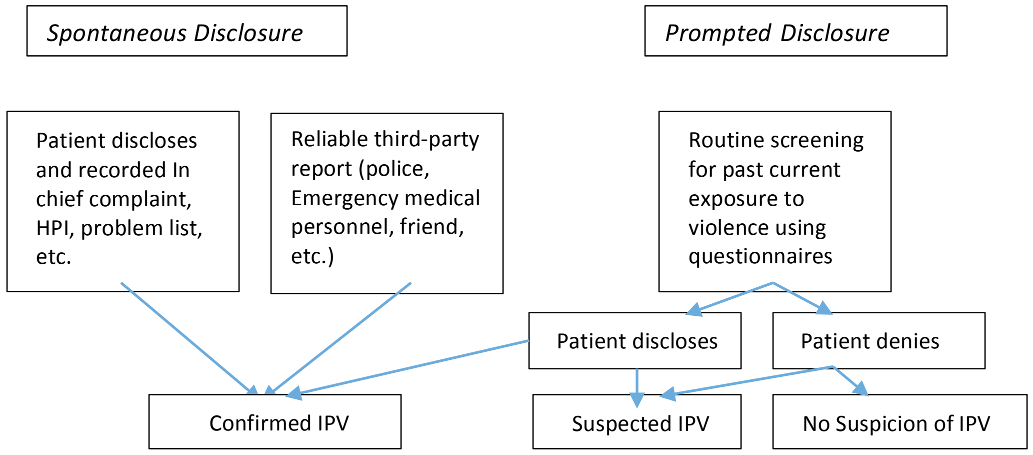

Figure 1. Operational model for diagnosis of IPV in clinical settings

C.3.1. *Informatics:*

The foundation of the approach is an informatics enabled systematic workflow for administering questionnaires for patient reported outcomes in clinics. Our goal is to embed within the EHR, as completely

as possible, effective procedures and methods for screening, assessment, tracking and referral of patients for counseling for IPV.

Figure 2 shows a block diagram of the IPV screening process. The goal is to implement, via the EHR, automated annual screening for IPV for an institution's female at risk population.

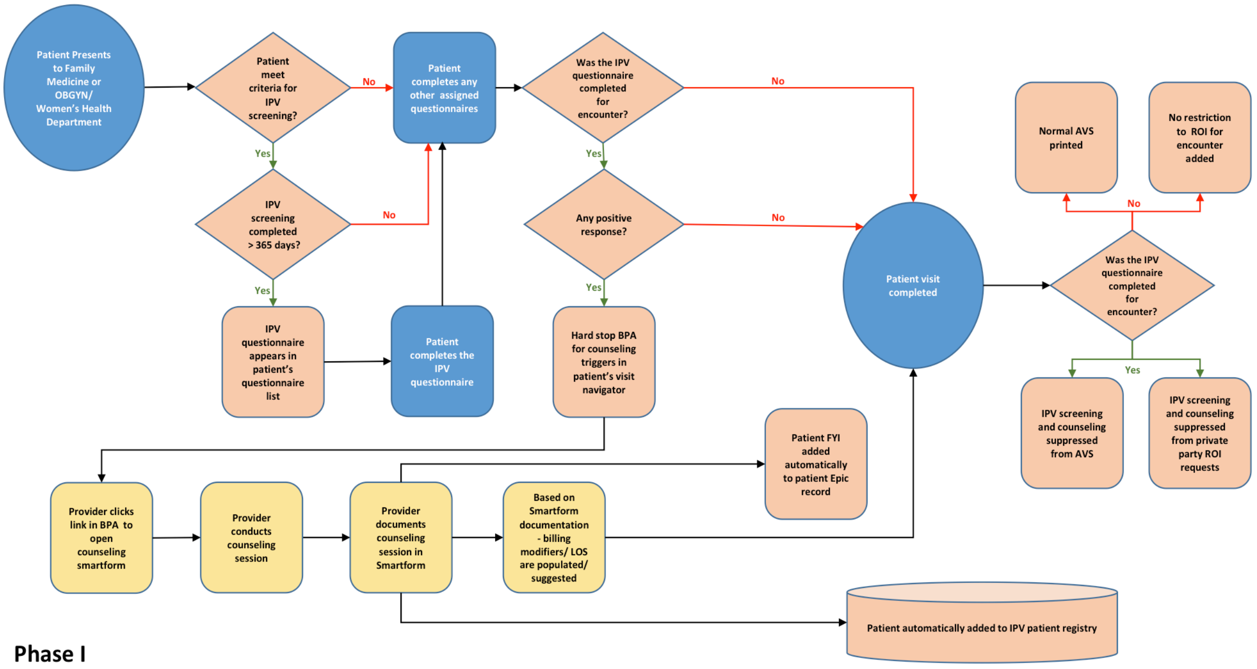

Figure 2. Design the IPV care system in Epic

As diagrammed, the EHR application automatically determines patient eligibility for screening, looks for past screening within the past 365 days and then adds the screener questionnaire to the patients' personal questionnaire queue. MUSC would modify existing Epic software used for research preferences and other PRO projects for this workflow.

Screening can occur at various places in a clinical encounter. For purposes of application development, we postulate that a *patient encounter* within the health system consists of four basic processes:

1) Registration (entry of arrival of the patient into the EHR and collection of billing and other data)
2) Rooming (vital signs, chief complain capture, other routine measurements by medical assistant or nurse)
3) Provider visit (primary care provider time in room with the patient)
4) Post visit care (ancillary provider visits (pharmacist, psychologist, etc.), review of visit summary, and scheduling of return visit.

Integrating a screening task with workflow requires either changing one of these four processes or adding new ones. The times between these events are potential places to introduce new processes that are patient led. In some settings, the time between registration and rooming is used with a tablet to administer a screening questionnaire. The nature of the IPV questionnaire and the potential for influence by family members, screening in the waiting room is not a safe or effective approach for IPV identification. Kiosks might offer more privacy but require space and resources. Also at MUSC and other institutions, there is an increasing trend toward immediate rooming of patients to expedite care. These issues make use of the waiting room problematic. Use of the provider's visit time (#3) to orally administer the screening questionnaire is probably inefficient and complicates providers' response to the patient's visit agenda Use of time after the provider visit (#4), is possible, but if a problem is detected, attention requires interruption of the provider's care for other patients, creating workflow issues.

Given these obstacles, at MUSC, we have developed a workflow for administration of PRO questionnaires during the rooming step. Questionnaires are administered between completion of rooming tasks (vital signs capture ect.) and the provider visit. The exam room provides privacy. As a policy, family members and older children can be asked to leave the room during questionnaire completion. As a default, questionnaires are self-completed by patients. This is important in IPV screening as automated screening questionnaires have been shown to increase IPV detection rates in general medical populations.(Klevens et al. 2012) Custom modifications of Epic support the safe use of the (physician's) exam room computer by patients for questionnaires, locking the provider's screen. This reduces costs for purchase and maintenance of equipment. However, if needed, our implementations will allow patients to use tablets to complete questionnaires (for example, if the exam room layout requires this.) Patients who are not proficient in computer use or speak other languages than English have questionnaires orally administered by medical assistants and/or translators. Another, novel already deployed modification to Epic, that will support IPV screening is a PRO "questionnaire queue" for patients, linked to a calendar. Questionnaires can be automatically added to a patient's queue based on the last completion date. PRO questionnaires are implemented in MyChart (Epic's patient portal tool) and the results from questionnaires are immediately available to decision support tools within Epic. After completion of the assigned questionnaires, the questionnaire tool is closed and the EHR work screen is locked, awaiting the provider's entry in the room.

The PRO questionnaire administration process is in production at MUSC for capture of patients' preferences for participation in research (about 50 clinics and about 6,000 administration of a 4-item questionnaire), for back pain (11 clinics and about 1400 patient completions. Extensions of the workflow are in pilot testing in HIV and oncology clinics. Given adequate training of staff and feedback, the process is highly acceptable both to staff and to patients.

If the patient screens positively for IPV, the EHR automatically detects this and triggers a mandatory pop-up style (in Epic parlance "Best Practice") alert that providers must acknowledge before closing a patient file. MUSC's Epic Research team produces these alerts on a routine basis for clinical trials. MUSC already has implemented patient questionnaires that are linked to provider documentation tools as part of PCORI back pain trial. Clicking on the alert for IPV takes the provider to a *Confidential Epic Department* (a standard separate section of the EHR that can only be accessed by authorized providers except in emergencies, and even in this setting, access is tracked.) The parts of the protocol occurring in the confidential Epic department are depicted in yellow in Figure 2. This specific task (creation of a note in a new section) requires extension of our current Epic tools. Using templated note forms and additional questionnaires, the provider would confirm the diagnosis, determine the risk of harm, and if needed initiate a referral to the 800 number for counseling and, if at high risk of harm, contact hospital social work and security services, if needed. The provider would perform this task during the routine visit, after asking family members and other accompanying persons to leave the exam room. The template (see mockup in Figure 4), would guide the provider's diagnostic interview, risk assessment, referral strategy and follow up while generating a text progress-note for documentation and

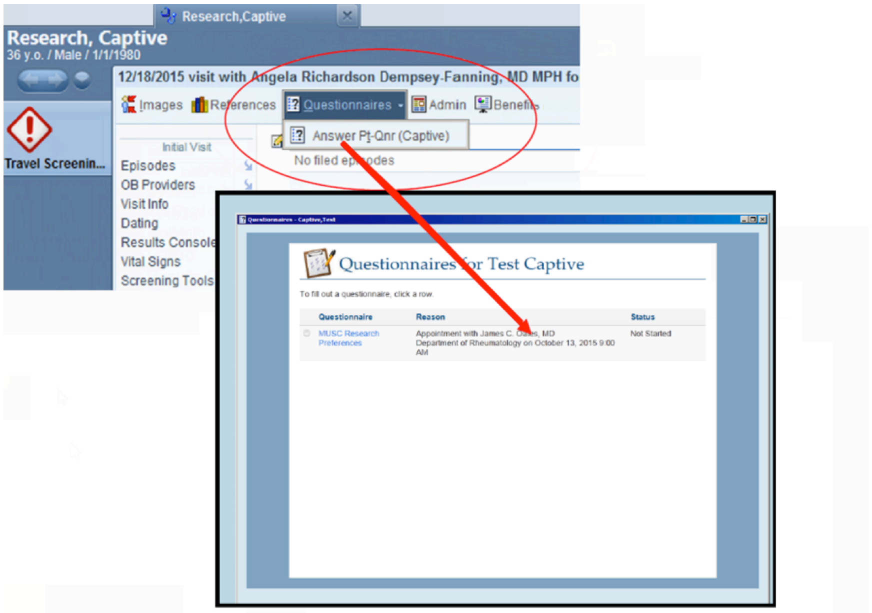

© 2017 Epic Systems Corporation. Used with permission.

Figure 3. PRO administration using the provider's workstation.

billing purposes. Software for templates that generate progress notes would be patterned after tools created by the AHRQ funded Neurology Practice Based Research Network that has been implemented at MUSC. Content would be taken from other tools for note writing created by NCKP and published on the Epic User Web site. (http:// userweb.epic.com)

After completion of the provider's part of the visit, but before the other post visit activities, there would be an automatic process for initiation of counseling via telephone or secure telehealth session using National Domestic Violence Hotline. The provider would connect the patient to the counselor using either a phone in the exam room, telehealth functionality built into Epic or, a HIPAA compliant commercial standalone system telehealth system for web browsers (doxy.me) delivered on a low cost external device such as a Chrome Book. The initial telephone/telehealth counseling session would be completed in the exam room or another room in the clinic when feasible. If a patient is deemed at very high risk, providers would immediately refer to health system social work. Subsequent sessions with the Helpline would occur by the patient's preferred method of communication. After completion of counseling, the patient would be checked out in the normal manner. IPV services would be billing out of the confidential department and would not appear on visit summaries. Providers would complete two notes—their regular progress note and an IPV care note in the confidential department. They would subsequently bill for both services planned for the visit and IPV care, but separately to preserve anonymity. RVUs for both the confidential department and usual care would be used to measure productivity. Providers would also capture information on whether the patient can be directly contacted for follow up and the best approach (telephone, text, cell, email) and number to do so. The study team will use this information to contact the patient and collect data on outcomes of treatment after consent.

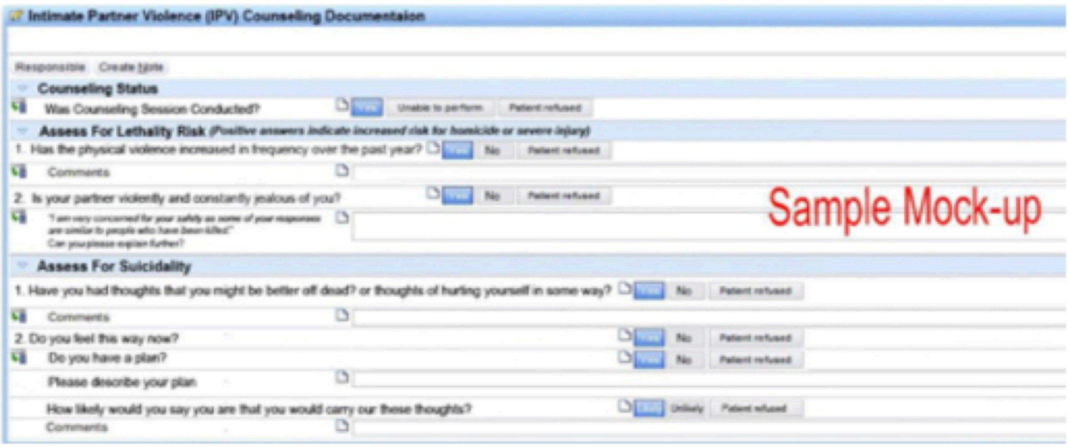

© 2017 Epic Systems Corporation. Used with permission.

Figure 4. Template for IPV examination and note generation by provider

The National Domestic Violence Hotline (Campbell, Webster et al. 2009, Chapin, Coleman et al. 2011) provides 24 hour/7 days-a-week access to counselors for IPV care across the United States. The Hotline was established in 1994 by President Clinton as part of the Violence Against Women Act. Over the past 22 years, the Hotline advocates has served over 3 million callers. Hotline service providers are payed employees trained in crisis counseling and receive ongoing training. The Hotline provides a wide range of services to both English and Spanish- speaking victims, as well as to friends and family of the victims and to abusive partners. Available services include, but are not limited to: assessment of safety concerns, assistance in developing safety plans, support via empathetic listening, assessment of victim needs, options and education regarding available resources (e.g., victim advocacy, shelter, and counseling), referrals for legal help, and education on technology and social media safety. The majority of first time hotline service calls involve basic information about safety options, support, and overview of potential types of resources. Hotline service providers have contact information of local resources, including shelters and details about specific shelters (e.g., space for children and/or pets). All counselors working for the hotline are extensively trained. There are strong internal processes for quality control. The hotline will report weekly on the number of patients who contact the hotline from each MUSC clinic. Use of the hotline ensures that when the intervention is further disseminated, the intervention is delivered with a high degree of integrity.

Patients who have or are experiencing IPV would be enrolled in a confidential IPV registry. Prior to the next visit, the provider would receive an Epic message notifying him of a scheduled visit by a patient who had screened positive for IPV or had been diagnosed with IPV. During the visit, the provider would again be reminded of the IPV care by a “non- blocking” pop up alert, which if clicked upon, would take the provider to the confidential department to document subsequent episodes of IPV care. This will help ensure follow up care, even if IPV is not named as an active problem on the patient’s problem list (to reduce the risk accidental disclosure to spouse or family member.)

The proposed workflow will handle all IPV care. If, as in Figure 1 on the left side, IPV is spontaneously disclosed by patient or third-party report in clinic, the provider would move manually to the confidential department for IPV risk evaluation and screening. The proposed workflow will be refined iteratively, using agile methods, working with staff and providers in one model clinic from each respective sub discipline of primary care (family medicine, general internal medicine, and gynecology). Documentation for billing will be reviewed by the MUSC compliance office. All work will be done by MUSC’s dedicated 6-person Epic Research team, led by Melissa Habrat under the supervision of Dr. Lenert. This is the same team that has developed MUSC’s PRO capture tools described above.

C.3.2. *Human factors/design:*
User interface design and human factors contribute to the success or failure of a system. Poorly designed user interfaces can increase cognitive load, decrease efficiency, confuse users, and lead to errors. Therefore, human-computer interaction and usability experts, Drs. Welch and Madathil (Welch’s mentor in his K award), will be involved throughout the planning, design, testing, and evaluation to ensure the user interface adheres to established design heuristics and usability best-practices. Welch and Medathil will refine established workflows such as the in-clinic PRO capture workflow. However, their primary focus will be on novel key workflows, such as IPV risk assessment/counseling, provider documentation, the warm handoff of the patient to national hotline. Billing procedures will also be studied. Testing will occur among a small sample of target end-users using low-fidelity prototypes and early functional versions of the solution. This will ensure the user interface effectively supports the intended goals of the project and minimizes risk of inadvertent confidential disclosures.

C.4. Aim 2: Deploy and evaluate the system in MUSC primary care and women health clinics

C.4.1 *Design*

This aim focuses on the implantation of the EHR tools linked to the National Domestic Violence Hotline across a health system. As a well-designed implementation research, we will implement the developed system as part of a stepped wedge cluster randomized trial (Hemming, Haines et al. 2015). The stepped wedge approach is a pragmatic design that is maximally acceptable to implement and evaluate the intervention (IPV screening, diagnosis and referral). Through this approach all participating practices will be exposed to the intervention, and the random allocation of start times is considered a fair approach to use to account for the fact that it is not practical to implement the intervention in all practices at once. It is also a statistically powerful approach that allows proper evaluation of the impact of the intervention as each clinic can serve as its own contemporaneous control.

	Q4-Q5	Q6-Q7	Q8-Q9	Q10-Q11
	6 Mos	6 Mos	6 Mos	6 Mos
Practice 1 to 4	2423	2422	2422	2423
Practice 5 to 8	2423	2422	2422	2423
Practice 9 to 12	2423	2422	2422	2423
Practice 13 to 16	2423	2422	2422	2423
Practice 17 to 20	2423	2422	2422	2423
Practice 21 to 24	2423	2422	2422	2423
Practice 25 to 28	2423	2422	2422	2423

Table 1 . Design matrix for Stepped Wedge study as described by Brown and Lyford (Brown and Lilford 2006).

Brown and LIlford (Brown and Lilford 2006) used a box figure to show the order of randomization in the “stair-step” pattern. Table 1 shows the number of visits in each time period. In our model, current best practices for screening for IPV represent the yellow control blocks. All clinics will receive the intervention after a baseline period. Over time, clinics are randomized to receive the EHR screening intervention to systematically screen

all women in the practice (pink blocks). The randomization will be conducted by the study biostatistician and will be stratified by two factors: Total Number of Clinic Encounters by the most relevant female age group (18-49) for IPV risk (Factor 1) and Type of Clinic (FM, IM, or GYN (Factor 2). First the clinics will be ordered by Factor 1 and divided into 4 subgroups (blocks), with each including 4 clinics. Clinics in each block will be further ordered by Factor 2, and assigned to active intervention start as first, second or third group, making sure that the groups will be balanced in terms of both Factor 1 and Factor 2.

C.4.2. *Usual care description:*

As recommended by IOM report and USPSTF, MUSC believes that all women should be screened and counseled for interpersonal and domestic violence as part of standard of care within primary care practice. Annual screening for IPV is supposed to be provided within MUSC primary care following general national guidelines, but there is currently no systematic approach to this screening within primary care practices. Current screening is provided in the form of self-report questionnaires and/or physician or nurse in-person follow-up. Physicians and employees within MUSC complete yearly online training on general information about domestic violence. In addition, an educational toolkit website housing provider resources, patient materials, and referrals was developed in 2007 as part of a Duke Endowment grant. (see http://academicdepartments.musc.edu/nursing/pcp/index.html). Within the EHR, IPV screening questions are available if physicians wish to choose suggested questions but are not systematically integrated into care. MUSC has an operational Domestic Violence Steering Committee that develops recommendations for IPV care across MUSC clinics. A recent output of this committee is a one hour online training course on working with IPV for professionals in the nursing, mental health, and medical fields. Several other one hour online trainings related to domestic violence are offered as electives to MUSC staff and faculty including: Implementing Routine IPV Screening in a Primary Care Setting and Safe Zone at MUSC.

C.4.3. *Intervention description:*

In Aim 2, we will randomize the MUSC primary care practices in groups of four practices with six months elapsing for each step in the randomization. There will be two steps for each practice: (1) Current best practice for a minimum of one step; (2) Best practice for questionnaire based screening, diagnosis, and referral using an EHR system (developed in aim 1. The research advantages to using the stepped wedge design is that each practice will provide data on screening and referral rates to the IPV hotline under the conditions of current best practice, as well as under conditions of EHR-supported screening.

C.5. Participants and sites:

The study will be implemented at MUSC Primary care and Women's Health practices. MUSC Health Primary Care provides services via 28 practices spread across three counties in South Carolina that employ over 40 Physicians (Family Physicians and General Internists) and 10 Nurse Practitioners and Physician Assistants. MUSC Women's Health (WH) provides care through five practices that employ over 20 gynecologists and nurse midwifes. The two WH sites with the largest primary care practice will be used. Although there are differences in the sites regarding staffing and size, all sites use the same EHR and have access to practice reports with data extracted from the EHR that evaluate the quality of care provided. Most sites have received recognition as a Patient Centered Medical Home (PCMH) through the National Committee for Quality Assurance (NCQA), and currently use EHR prompts to provide guideline-based screening, although no prompts are currently available for IPV screening. Approximately half of the patients served are African American or Hispanic, which is consistent with the demographics of the area served. The payer mix for the practices includes about one-third Medicare and one-fifth Medicaid insurance.

Certain patient subgroups are of particular interest because they have been reported to be of greater risk for and it is not known if counselling is more or less acceptable or effective for these groups (Black, Basile et al. 2010); Black and colleagues report that relative lifetime risk of IPV is 1.26 for black women and 1.09 for Hispanic women when compared to whites. Further, risk of IPV is highest in women under age 25. We will have adequate enrollment of black women and young women to have statistical power to conduct subgroup analyses of patients in these high-risk subgroups to examine important characteristics of the intervention. We will not be able to examine difference for Hispanic, Asian, or Native American women because of low

representation of these groups in SC. We will also explore difference in women living in rural communities and in low income geographic areas by using patients' home address to geocode and construct such measures for the study population. Differences in rates of screening refusal, rates of positive response on the screening questions, variations in types of IPV responses, acceptance of counselling and use of the Hotline will be examined by sub-groups defined by age [18-25 vs. 26-49 years; black vs. non-black race; rural vs non- rural census tracts; and low-income census tracts (to be defined)].

C.6. Formal protocol development:

Working with stakeholder representatives, we will develop a formal study protocol specifying the purpose for which data will be collected (comparative effectiveness as fully described in the evaluation plan), data sources and linkage plans, data feasibility and quality, and measures of effect. During Project Year 1 , we will develop a formal study protocol that includes the following core elements: (1) Design for Epic modifications, including screening questionnaires, diagnostic interview support, referral order set and smart phrases, and "hidden" department documentation of counseling and billing; (2) Specification of workflow and standard operating procedures (SOPs) for screening, diagnosis and referral in practical clinic settings; (3) Specification of workflow and SOPs for hotline staff; (4) Manualized training materials for clinic physicians and staff regarding use of Epic software and screening tools, as well as manualized Helpline training materials regarding workflow, software documentation tool modifications, and operations modifications; (5) Data extraction protocol specifying fields in Epic to be extracted and mapping to Research Data Warehouse, an SOP for extraction and insertion of patient data from interviews into REDCap, an SOP for extraction from REDCap and integration with EHR data for analytic file production, and a data dictionary; and (6) Data analysis plan.

C.7. Comparison groups:

The two main comparators in the current study are current best practices for screening for IPV (control) and EHR supported IPV screening, diagnosis, counseling, referral, and billing (intervention). Our control condition was selected based on current guidelines for IPV screening and follow-up care. Our intervention condition was selected, consistent with findings from the NCKP approach (Miller, McCaw et al. 2015), given its evidence-based potential to leverage EHRs as an additive support for the implementation of the best practices in IPV care that constitute our control condition. The stepped wedge cluster randomized design of the study will allow us to isolate the causal effects of EHR support on outcomes of interest (e.g., IPV disclosure, referral, practitioner and patient satisfaction) by allowing each cluster of clinics to provide observations for both the control and the intervention conditions. In addition, this study design will reduce potential for bias introduced by controlling for contamination effects across clinic staff in a given setting.

***C*.8. Recruitment, randomization and retention:**

To recruit clinics for participation, we will work with the Primary Care Service Line Director, clinical leaders in Internal Medicine, Gynecology and Family Medicine and staff to implement the study. Prior to roll out in each clinic, we will develop and train relevant clinic staff and providers using the following tools and approaches: 1. Manual for clinic operations of the system 2. Online training for helpline 3. Pre-intervention in-service, online education, and certification for clinic staff and providers. Online training will be integrated with MUSC's MyQuest staff education system. Completion will be mandatory but incentivized ($5 Starbucks card) and rates monitored. During the initial week of the roll out, Epic research support personnel will be available in the clinics to help providers learn to use software modifications and to answer questions. Provider support for in-person individual IPV-related training and case consultations will be provided by two experts in IPV training and management through-out the implementation and operation of the study.

This study is a clinic-level stepped wedge randomized trial. The major sources of potential bias in data collection within a cluster-RCT design include 1) inappropriate selection of sites, and 2) inadequate data collection once subjects are enrolled within a site (Campbell 2014). To address issue #1, clinics will be stratified and randomized in blocks of four. The randomization will be conducted by the study biostatistician and will be stratified by two factors: Total Number of Clinic Encounters by main age risk group (Factor 1) and Type of Clinic (FM, IM, or GYN (Factor 2). First the clinics will be ordered by Factor 1 and divided into 4 subgroups (blocks), with each including 4 clinics. Clinics in each block will be further ordered by Factor 2, and

assigned to active intervention start as first, second or third group, making sure that the groups will be balanced in terms of both Factor 1 and Factor 2. To address issue #2, EHRs of all participating clinics will be analyzed every two weeks for screening failures, refusals and data completion rates. De-identified aggregate data completion rates will be provided to the individual clinics through the site champion on a biweekly basis. In person meetings with the site staff to discuss data collection rates for the previous month and cumulative-to-date will be reviewed for each clinic and discussed with staff as a whole as a measure of quality improvement. This approach will allow rapid identification of potential barriers to screening, referral and data collection and rapid and relevant quality improvement interventions to address barriers. Distributions of baseline characteristics for clinics and patients will be compared between the control condition and the active screening condition to assess effectiveness of the randomization, and statistical or clinical differences will be adjusted for in sensitivity analyses. All analyses for treatment group comparisons will use an intention-to-treat approach.

Table 2. Recruitment plan

1. Estimated number of potentially eligible study participants: *The number of study participants were estimated based on the number of unduplicated females seen in the 28 primary-care and OB practices during 12 months from 10/1/2015 through 9/302016.*	71,400
2. Total number of study participants expected to be screened: *We expect **33,915** to receive current practice treatment and **33,915** to be offered IPV screening.*	67,830
3. We *expect a 95% screening acceptance rate based on reports on acceptability in the literature. The US rate of IPV during 12 months reported in the most recent survey is 5.9%. The US-South Carolina RR of lifetime IPV is 1.17. Thus, we expect that 6.9% of screened women will have experience IPV during the last year and that at least 80% will provide a positive response to our screening instrument. Thus a 5.5% IPV rate is expected during the active intervention times.*	32,219
4. Target sample size : *We expect that a minimum of 75% of women who screen positive will accept counselling in the office and that 50% will contact the Hotline during the active phase of the study*	1,329 and 886
5. If applicable, total number of practices or centers that will enroll participants: *14 Family Medicine, 12 Internal Medicine Primary Care and 2 women's health clinics*	28
6. Projected month first control group data extract (month after project initiation):	9 months
7. Projected month last active participant enrolled (month after project initiation):	33 months
8. Projected rate of enrollment (anticipated number enrolled 33,912 over the 12 -month active enrollment period): *These are the expected number of women screened per month*	2,423
9. Estimated percentage of participant dropout: *We do not expect drop outs because the study is a one-time screening. However, we do expect refusals and for subjects who decline counselling.*	NA

C.9. Procedures of data collection and management:

The patient-level data for this study are linked within the EHR and REDCap (used to capture data for follow up interviews). Data on clinic characteristics will be merged onto the analytical data set using the clinic identifying number and manually checked for validity. A research data honest broker will use this to create anonymized files free of HIPAA identifiers (MRN converted to a study ID, dates will be shifted to relative dates, birthdate to age, etc. Social demographic factors will be obtained by geolinkage. An unduplicated list of patient census tract codes will be used to extract data from public use files on urban-rural status, indicators of poverty (e. g. percent of population below federal poverty limits, median income) will be constructed. These variables will then be merged onto the analytical data sets after the data has been extracted from the EHR. Standard data quality assurance approaches will be used to assure valid assignments. Missing values will be identified and treated as discussed in the evaluation plan.

C10. Analytic Plan: Measurements and sample size calculations are detailed in the *Evaluation Plan*

C.11. Future directions and dissemination plans:

The use of a nationally widespread EHR system (EPIC) and the integration of population health and billing

tools as well as leveraging the counseling and referral resources of the National Domestic Violence Hotline to overcome potential regional IPV resource barriers and thereby enhance scalability of this project. IPV screening in primary healthcare settings has been endorsed by numerous professional associations including the IOM , USPSTF the World Health Organization , American College of Obstetricians and Gynecologists , American Academy of Family Physicians, American Nurses Association, and the American Academy of Pediatrics. We will engage these organizations to disseminate results. To aid in future dissemination, we would study how to adapt ongoing efforts to other EHR platforms, including Cerner and Meditech, focusing on developing standards such as the Fast Healthcare Interoperability Resource protocol.

To support in early adoption of tools, all software programming and training materials will be available on request to any Epic installation. Working with Epic, will we will make a toolkit available on the Epic User Web (see letter) with including training materials within 18 months of the start of the proposal. In addition to presentations at the usual academic meetings, we will submit proposals for Epic Expert (XGM) and User Group (UGM) meetings (annual meetings that host thousands of clinicians and IT experts). If successful, we will work with Epic to make this installation part of its base system.

We will work regionally on direct dissemination through Health Sciences South Carolina, a regional learning health system collaborative, to encourage its members that use Epic systems (Greenville Health, Anmed Health, Self-Regional Health, and Spartanburg Health systems) to implement this screening program. Health Sciences also works with Duke, UNC Chapel Hill and Wake Forrest universities through the Carolinas Collaborative (CC), which has a shared data system spanning these universities and pilot funding mechanisms for cross institutional research to support dissemination efforts. All of the CC sites use the Epic EHR and could be part of a future scaling study. We will also work with Dr. Stern to take innovations to the North Carolina Coalition against Domestic Violence, which is working on EHR implementations of IPV screening for smaller hospitals and Dr. Brigid McCaw to bring refinements of the NCKP program developed herein back to NCKP and to other Kaiser Permanente regions. To help adopters implement and sustain implementations, we will have a monthly conference call, to provide ongoing support for implementation. MUSC is also launching an external fee-for-service Epic research consultation service. This team will be able to provide direct support for implementation of the software and programs developed.

Table 3. Timeline

	**Q1**	**Q2**	**Q3**	**Q4**	**Q1**	**Q2**	**Q3**	**Q4**	**Q1**	**Q2**	**Q3**	**Q4**
Activity	**Year 1**				**year 2**				**year 3**			
Clinic experience with IPV inventory (Baseline assessment in each clinic's current experience/use of IPV screening)	**X**					**f**						
Epic programming (*C.3*)	**X**	**X**	**X**	**X**								
Randomization (Block assignments) (C.4.1))				**X**								
Development and testing of Hotline call and tools for the "warm handoff"		**X**	**X**	**X**								
Manual development, pilot testing (C.3.1))		**X**	**X**	**X**								
**Control data collection** active				**X**	**X**							
**First clinic block active** (Launch of active screening in first 8 clinics (C.4.1)						**X**	**X**					
Clinic monitoring for uptake, satisfaction					**X**	**X**	**X**	**X**	**X**	**X**		
Clinic meetings (Feedback from advisory report discussions with each block of clinics)						**X**		**X**		**X**		
**Second clinic block active** (Launch of active screening in second 8 clinics) (C.4.1)								**X**	**X**			
**Third clinic block active** (Launch of active screening in the last 9 clinics) (C.4.1)										**X**	**X**	
Build of analytical data set											**X**	**X**
Final Report (Final report submission)												**X**

PHS Human Subjects and Clinical Trials Information

OMB Number: 0925-0001 and 0925-0002

Expiration Date: 03/31/2020

Are Human Subjects Involved ● Yes ❍ No

Is the Project Exempt from Federal regulations? ❍ Yes ● No

Exemption Number ❑ 1 ❑ 2 ❑ 3 ❑ 4 ❑ 5 ❑ 6 ❑ 7 ❑ 8

Other Requested Information

Human Subject Studies

Study#	Study Title	Clinical Trial?
1	EHR-based screening and intervention for intimate partner violence	Yes

Section 1 - Basic Information (Study 1)

OMB Number: 0925-0001 and 0925-0002

Expiration Date: 03/31/2020

1.1. Study Title *

EHR-based screening and intervention for intimate partner violence

1.2. Is this study exempt from Federal Regulations *	❍ Yes	● No

1.3. Exemption Number ❑ 1 ❑ 2 ❑ 3 ❑ 4 ❑ 5 ❑ 6 ❑ 7 ❑ 8

1.4. Clinical Trial Questionnaire *

1.4.a. Does the study involve human participants?	● Yes	❍ No
1.4.b. Are the participants prospectively assigned to an intervention?	● Yes	❍ No
1.4.c. Is the study designed to evaluate the effect of the intervention on the participants?	● Yes	❍ No
1.4.d. Is the effect that will be evaluated a health-related biomedical or behavioral outcome?	● Yes	❍ No

1.5. Provide the ClinicalTrials.gov Identifier (e.g. NCT87654321) for this trial, if applicable

Section 2 - Study Population Characteristics (Study 1)

2.1. Conditions or Focus of Study

- Intimate Partner Violence

2.2. Eligibility Criteria

Female Gender

2.3. Age Limits	Min Age:	18 Years	Max Age:	50 Years
2.4. Inclusion of Women, Minorities, and Children	2_4_Inclusion_of_Women_Minorities_and_Children1036155720.pdf			
2.5. Recruitment and Retention Plan	2_5_Recruitment_and_retention_plan1036155722.pdf			
2.6. Recruitment Status	Not yet recruiting			
2.7. Study Timeline	2_7_Study_TImeline1036155723.pdf			
2.8. Enrollment of First Subject	02/01/2019	Anticipated		

2.4 Inclusion of Women, Minorities and Children

The patient population of this trial focuses exclusively on women. We recognize that IPV occurs in men (but at a lower prevalence.) Evaluation of the impact of the screening procedure in men would require a much large study given its lower prevalence. Further, screening is not recommended for men at this time by the US Preventative Services task force, where it is for women. Therefore, we have, primarily for pragmatic reasons, limited the scope of this study to women.

This application does not include children since they are not at risk of intimate partner violence (IPV).

2.5 Recruitment and Retention Plan

This study is a cluster randomized trial in which primary care clinics at MUSC are randomized in sequence to begin computer screening for IPV, assessment of risk and referrals. Recruitment in this setting focuses on getting clinics to adopt the intervention into their routine workflows. To roll out the intervention, we will work with the Primary Care Service Line Director, clinical leaders in Internal Medicine, Gynecology and Family Medicine and staff to implement the study. Prior to roll out in each clinic, we will develop and train relevant clinic staff and providers using the following tools and approaches: 1. Manual for clinic operations of the system 2. Online training for helpline 3. Pre-intervention in-service, online education, and certification for clinic staff and providers. Online training will be integrated with MUSC's MyQuest staff education system. Completion will be mandatory but incentivized ($5 Starbucks card) and rates monitored. During the initial week of the roll out, Epic research support personnel will be available in the clinics to help providers learn to use software modifications and to answer questions. Provider support for in-person individual IPV-related training and case consultations will be provided by two experts in IPV training and management through-out the implementation and operation of the study.

Retention in the setting of a cluster randomized trial of a single point in time intervention will focuses on continuation of the intervention in the clinicsafter the initial adoption period. EHR data from all participating clinics will be analyzed every two weeks for screening failures, refusals and data completion rates. De-identified aggregate data completion rates will be provided to the individual clinics through the site champion on a biweekly basis. In person meetings with the site staff to discuss data collection rates for the previous month and cumulative-to-date will be reviewed for each clinic and discussed with staff as a whole as a measure of quality improvement. This approach will allow rapid identification of potential barriers to screening, referral and data collection and rapid and relevant quality improvement interventions to address barriers. Distributions of baseline characteristics for clinics and patients will be compared between the control condition and the active screening condition to assess effectiveness of the randomization.

2.7 Study Timeline

	Q1	Q2	Q3	Q4	Q1	Q2	Q3	Q4	Q1	Q2	Q3	Q4
Activity	**Year 1**				**year 2**				**year 3**			
Clinic experience with IPV inventory (Baseline assessment in each clinic's current experience/use of IPV screening)	X					f						
Epic programming (*C.3*)	X	X	X	X								
Randomization (Block assignments) (C.4.1))				X								
Development and testing of Hotline call and tools for the "warm handoff"		X	X	X								
Manual development, pilot testing (C.3.1))		X	X	X								
**Control data collection** active				X	X							
**First clinic block active** (Launch of active screening in first 8 clinics (C.4.1)						X	X					
Clinic monitoring for uptake, satisfaction					X	X	X	X	X	X		
Clinic meetings (Feedback from advisory report discussions with each block of clinics)						X		X		X		
**Second clinic block active** (Launch of active screening in second 8 clinics) (C.4.1)								X	X			
**Third clinic block active** (Launch of active screening in the last 9 clinics) (C.4.1)										X	X	
Build of analytical data set											X	X
Final Report (Final report submission)												X

Inclusion Enrollment Reports

Entry#	Enrollment Location Type	Enrollment Location
Study 1, IER 1	Domestic	Domestic

Inclusion Enrollment Report 1

Using an Existing Dataset or Resource* : ❍ Yes ● No

Enrollment Location Type* : ● Domestic ❍ Foreign

Enrollment Country(ies): USA: UNITED STATES

Enrollment Location(s): Domestic

Comments:

Planned

Racial Categories	Ethnic Categories				Total
	Not Hispanic or Latino		Hispanic or Latino		
	Female	Male	Female	Male	
American Indian/ Alaska Native	0	0	0	0	0
Asian	13	0	1	0	14
Native Hawaiian or Other Pacific Islander	0	0	0	0	0
Black or African American	399	0	16	0	415
White	810	0	53	0	863
More than One Race	0	0	0	0	0
**Total**	1222	0	70	0	1292

Cumulative (Actual)

Racial Categories	Ethnic Categories									Total
	Not Hispanic or Latino			Hispanic or Latino			Unknown/Not Reported Ethnicity			
	Female	Male	Unknown/ Not Reported	Female	Male	Unknown/ Not Reported	Female	Male	Unknown/ Not Reported	
American Indian/ Alaska Native										0
Asian										0
Native Hawaiian or Other Pacific Islander										0
Black or African American										0
White										0
More than One Race										0
Unknown or Not Reported										0
**Total**	0	0	0	0	0	0	0	0	0	0

Section 3 - Protection and Monitoring Plans (Study 1)

3.1. Protection of Human Subjects	3_1_Protection_of_Human_Subjects1036155724.pdf
3.2. Is this a multi-site study that will use the same protocol to conduct non-exempt human subjects research at more than one domestic site?	❍ Yes ● No ❍ N/A
If yes, describe the single IRB plan	
3.3. Data and Safety Monitoring Plan	3_3_Data_safety_monitoring_plan1036155719.pdf
3.4. Will a Data and Safety Monitoring Board be appointed for this study?	❍ Yes ● No
3.5. Overall structure of the study team	3_5_Overall_structure_of_the_project_team_1036155864.pdf

3.1 Protection of Human Subjects

This program is designed to work at a clinic/institutional level to enhance IPV care through administration of a screening questionnaire, tools for assessment of risk and counseling, and tools for ensuring an effective handoff to counseling services. The primary risk to patients from the study is accidental disclosure of IPV screening results to the person abusing the patient. Great care is being taken to minimize the risk of this event. The screening portion of the study is a nationally recommended practice and subsequent tools are designed to help ensure appropriate care by primary care providers in clinical settings. Therefore, we will seek a waiver of consent from our IRB for patient participation the screening, evaluation and treatment referral protocol. If patients had to consent to be entered into the screening protocol, this would take more time than the screening activity itself, creating a substantial barrier that would not be there in practice, and making it impossible to evaluate the true impact of the protocol on care processes. This study could not be conducted if consent was required for screening, nor is it logical to do so, given that screening is a routinely recommended practice. We do seek consent for obtaining follow up data, as described below, using an approach designed to minimize the disruptive effects of research on clinical practice.

The study is designed to ensure beneficence and a positive risk to benefit ratio. Screening, assessment and referral interventions are known to be efficacious in helping patients get into counseling and respond to IPV threats. Evaluation of risk by the provider and referral are integrated into the screening program preventing issues with handoffs. Follow up care using registry and alerts to allow other authorized providers to re assess and respond to risks are also integrated. The study uses well-resourced national hotline with 24/7 availability and highly trained counselors with access to local resources. A local hotline might have better access to neighborhood resources, but use of the national hotline makes the insures availability 24/7 promotes the *fidelity* of the intervention and *reproducibility* of research results. Screening, the provider diagnostic interview, and the initial counseling sessions are all conducted within a clinic visit, in private, without family members being present in the room.

The study is designed to minimize risks of harm from accidental disclosure. To prevent accidental disclosure, physician risk assessments and other notes will be documented in a confidential department of Epic, so that documentation is protected against disclosure in medical record requests and patient downloading of CCDs. Only authorized providers will be able to see the department and review notes. The proposed IPV registry will be also maintained in a confidential department. Another risk for disclosure is accidental inclusion of IPV diagnosis in billing information. Billing for IPV care will be deferred or altered to prevent accidental disclosure in insurance claims, while preserving tracking RVUs for internal assessments of physician performance. Routine for medical records requests and CCD visit summaries will suppress IPV care information based on the information being documented in a confidential department. Legal requests for records will be carefully reviewed for evidence of coercion of the patient and appropriate responses taken should evidence arise.

The Epic Resarch Team at MUSC who will make the modifications to the HER is highly experienced in addressing confidentiality issues. MUSC maintains multiple confidential departments in its Epic instance, including a department for crime victims that supports the National Crime Victims Center (co-led by Dr. Rheingold). MUSC also has experience administering studies with HHS Certificates of Confidentiality in Epic. This approach was considered for this study but deemed impractical (The approach used--a second patient ID in Epic—designed for substance abuse studies, would impair longitudinal care and have its own risks of disclosure to spousal abusers ("What is this second MUSC medical ID card about and who is Jane Doe?".)

Another potential risk area is assessment of patient satisfaction and acceptance of IPV care process and assessment of follow up outcomes in telephone interviews. Permission to contact will be captured as part of the screening process. There are critical safety considerations when collecting research data from victims of crime, particularly those who have experienced assault from an intimate partner. Fortunately, the faculty at the National Crime Victims Center (including PI Dr. Rheingold and Co-I Moreland) have extensive experience working with victims of intimate partner violence. They both are licensed clinical psychologists who would be available for crisis intervention if need be. They routinely utilize the safety protocol specified by Sullivan and Cain (2004) when collecting data from this high-risk group. This same protocol will be used in the current study to minimize the risk of harm to participants.

Research assistants will be trained to follow a structured safety protocol when collecting research data from participants who have experienced intimate partner violence. The protocol is fully specified by Sullivan and Cain (2004), and the key steps are outlined in Human Subjects Table 1 below.

Table 1: Basic Safety Steps When Conducting Research with Victims of Intimate Partner Violence

1. Safety steps when first contacting potential research participants by telephone.

If a man answers:

• Ask for the woman by first name.

• If he asks who you are, provide very little information and state that you are calling about a research survey.

If a woman answers:

• Don't assume it's the woman you're looking for.

• Ask for the woman by first name.

• Ask if this is a good time to talk.

• Listen for verbal cues that she might be unsafe or frightened.

By mail:

• Assume others might read the mail.

• Do not mention the details of the study.

2. Safety steps when conducting in-person interviews. Interviews not occurring at the woman's home (for example, during follow up clinic visits):

• Conduct the interview in a location that is well-lit and secure. Make sure a cell phone is available.

Interviews occurring at the woman's home:

• Stress that no other adults can be present during the interview for any reason.

• Have a "story" in place in case the intimate partner interrupts the interview. One option is to state that you are conducting an interview pertaining to women's health (be sure to have a fake health survey on hand). Importantly, the researcher and participant should develop an agreed upon "story" prior to the interview.

• Be prepared to stop the interview and continue at another time if safety is compromised.

• Let the participant know that if the assailant comes to the home during the interview, it will be ended or postponed. Ask the participant if she has any concerns about the partner's behavior and if she thinks the partner would be upset if s/he saw the interviewer. Ask the participant if she would like the police called if the assailant comes to the home.

3. Safety steps when contacting participants over time.

• Have participants sign Release of Information forms allowing family members and/or friends to notify researchers about any changes in the participant's residence or phone number. The form should simply state that the participant is involved in a research study but should not provide details about the study.

• Write down times and places it is safe to contact the participant as well as times and places it is unsafe.

• Ask if it is safe to leave messages on the participant's answering machine or voice mail, but even if it is, never mention the nature of the study in the message.

• Prior to conducting phone-based interviews, develop a code word that the participant can use to let researchers know if it is an okay time to talk.

• Make sure participants have a safe and easy way to notify researchers if they change their minds about participating for any reason.

• Assume that some perpetrators will obtain the research's telephone number and make sure all project staff who might answer the phone are trained to deal with this safely. Staff should not share the true nature of the study with anyone who calls, and there should be a generic study name that staff members use to refer to the study (e.g., Women's Health Study).

• If the research office has voice mail or an answering machine, the message should be vague and should not refer to violence or abuse.

4. Additional general safety considerations.

- *Researchers should never assume a participant is at low risk of being abused simply because she is no longer in a relationship with the perpetrator.*
- *Researchers should be prepared to offer participants written information about local domestic violence service programs and community resources.*
- *All research study staff that might have contact with study participants will be trained in basic safety planning.*
- *Types of questions to ask participants if safety is an immediate concern:*
- *Are you safe right now?*
- *Do you want me to call the police for you?*
- *If we get disconnected, I'm going to call the police, okay?*
- *Do you have a personal protection order in place?*
- *Where could you go to feel safer?*
- *Can I give you the number of the local domestic violence shelter program?*

For participants enrolled in the follow-up interview portion, in any circumstance where a research assistant is concerned regarding potential for harm to self or others, Dr. Rheingold will be contacted immediately. In rare instances where Dr. Rheingold cannot be reached, Dr. Moreland will serve as a back-up. In circumstances where participants express suicidal ideation and are sincerely willing to contract for safety (i.e., will agree to keep her/himself safe), a plan will be made and carefully monitored. If a participant is deemed at risk for suicide and will not contract for safety or has made a suicidal gesture or attempt, the participant will be assessed by a local emergency department for possible inpatient hospitalization. Drs. Rheingold and Moreland have the capacity to arrange for direct inpatient admission assessment at the MUSC inpatient units (i.e., the MUSC Institute of Psychiatry) for participants requiring such assessment (e.g., concerns about danger to self or others). Dr. Rheingold also will ensure that participants that present with risk for harm are well educated on how to access 24- hour emergency care (through 911 or going to the local Emergency Department).

To protect patient confidentiality during the analysis phase, a study data will be extracted from Epic. Coded identifiers will be assigned by an honest broker in the MUSC Research Data Access team. The broker will create an integrated file that censors HIPAA personal data. Dates will be abstracted to step periods and time deltas, date of birth to age, etc. Linkages will be stored in an encrypted file that only the honest broker can access. The analytic file will be stored on password protected servers that only study team members can access.

3.3 Data safety monitoring plan

The primary risk of this study is to patients from inadvertent disclosure of reporting of IPV or of perceived threat of IPV from the spouses' partner. To monitor for issues, we will institution a screening program for adverse events related to use of IPV software. As part of the program, we will on a bimonthly basis:

1. Electronically survey providers by EHR messaging in clinics asking them about incidents in clinics from patients' spouses or patient reported events in subsequent visits.
2. Review responses of patients in telephone surveys and identify instances where screening may have led to adverse events.
3. Create Epic reports identifying patients that have admissions to ED or hospital with trauma within 1 month of IPV screening.

Survey responses and Epic reports will be monitored by the study PIs. The PIs will interview the providers and, if it can be performed safely, patients, to determine if screening procedures led to disclosures that resulted in adverse events. If this is found to be true, the intervention will be halted and software revised to issues related to disclosures.

3.5 Overall structure of the study team

The study is organized as a dual PI study, with Leslie Lenert MD being the administrative lead and focusing on work on Informatics and EHR modification and Alyssa Rheingold, PhD being the lead subject matter expert on IPV. The team is enhanced by co investigators with special expertise in human factors engineering (Brandon Welch) and in the psychology of trauma (Jenna McCauley). Vanessa Diaz, MD the Clinical Services Director for the Department of Family Medicine supports clinical integration activities. Kit Simpson, DrPH supports the analytics. Azza Shoaibi is a post doctoral fellow in informatics supporting the project. Ms. Cheryl Samuels is the project manager. Melissa Habrat is the lead analyst for Epic Research at MUSC. Epic programmer TBD is a composite of the Epic Research team assets (5 members in addition to Ms. Habrat) that will, as a team, support Epic development efforts. Patient stakeholders, national advocacy leader, and the original developer of the Kaiser System, all serve as consultants to the project. Counseling services are provided under contract to a national hotline.

The project is conducted at MUSC in primary care clinics staffed by the university. A total of 28 primary care clinics will participate in the study that are a mix of Family Medicine, Internal Medicine, and Women’s Health primary care settings.

Section 4 - Protocol Synopsis (Study 1)

4.1. Brief Summary

Violence against women in the US is a serious public health problem and a human rights issue. The U.S. Bureau of Justice Statistics reports that 22% of violent crimes against women are accounted for by Intimate Partner Violence (IPV). IPV screening recommendations support universal screening to include all women and not only those with obvious signs of physical abuse. However, the overall prevalence of screening for intimate partner violence (IPV) in health care settings is still low. Moreover, evidence on the effectiveness of screening programs are conflicting. Recent studies indicates that, while screening increases the rate of identification of IPV, it has not been shown to increase rates of referral for IPV counseling or improve health outcomes. Evidence also indicates that effective IPV screening programs are those with a “systems level” approach to design and implementation. The lack of compelling evidence on the effectiveness of IPV screening programs might be related to lack of the system level changes required for such programs to succeed. This study aims to fill the knowledge gap by designing, implementing and evaluating a health system level intervention through EHR modifications to improve IPV care. The proposed intervention includes several elements: tools and decision support for screening and detection of IPV, referral to national IPV counseling resource via telephone/telehealth and EHR modifications to support safe billing for IPV services. For screening and detection of IPV cases, the intervention focuses on routine inquiry by EHR embedded self-reported questionnaires and by an automated analysis of past records. These two sources are considered complementary to support providers with data to make timely decision about IPV diagnosis and subsequent referral. The two aims are carefully designed to accomplish all elements of the study.
Aim 1 focuses on developing an EHR-based decision support system for screening, diagnosis, referral and handoff of IPV care to a helpline. The system will use self-reported questionnaires as a routine tool to screen and detect IPV cases.
Aim2 focuses on implementing this decision support system through a stepped wedge cluster randomized design across 28 primary care clinics including family medicine, internal medicine and gynecology. The primary outcome of this aim is the rate of diagnosis by the provider of high IPV risk coupled with referral for IPV counselling to the National Domestic Violence Hotline or (in more extreme cases) to social work/security.
Secondary outcomes include the proportion of patients eligible that are screened, proportion of patients who are screened positive, proportion of screener positive patients that have IPV diagnoses confirmed through provider interview. On a clinic by clinic basis, we will also examine the time from registration to delivery of the after-visit summary for patients, the time for completion of the questionnaire, and the time for completion of a provider counseling session. In addition, we will study effect on RVUs overall and RVUs specific to IPV care from Epic. We will also measure provider and clinic staff satisfaction with Epic modifications and the counseling and referral process. In addition, we will determine the number of subsequent (to the episode of care in which IPV or IPV risk was identified) episodes of provider assessment and counseling and describe other usage of the registry by provider staff.
Patient centered outcome measures will be collected as a secondary outcome measure in patients who agree to be contacted by telephone by the study team (during their clinical visit as recorded at the end of the screening questionnaire) and who subsequently provider oral consent. In patients diagnosed with ongoing or risk of IPV, calls will capture data on: the number of contacts with the Hotline; satisfaction and acceptance of screening and intervention by the physician; consequences of screening; satisfaction with hotline services; help seeking activities (e.g., follow-up contact with Hotline, local advocacy/ shelter, mental health counseling); service utilization; posttraumatic symptoms; anxiety, depression and somatic symptoms; problematic drug and alcohol use: actions taken as a result of counseling; and finally any injuries in the interim and whether those injuries required medical treatment.

4.2. Study Design

4.2.a. Narrative Study Description

This study proposed to develop and validate an integrated suite of EHR tools that automates “best practices” from Northern California Kaiser Permanente for screening and intervention for IPV identified within clinical settings. The goal of the intervention goes beyond mere screening of patients for IPV and includes tools for assessment of IPV risk, for documentation of IPV care, and support for a warm hand-off during the primary care visit to a national IPV hotline resource. Importantly, the study includes work to keep notes documenting IPV and billing for IPV care confidential, segmenting this care in a secure segment of the electronic health record. The study uses a proven workflow for administration of Patient Reported Outcomes questionnaires in clinic developed at MUSC. It combines this work with tools for assessment of risks and documentation of counseling. A warm handoff will be achieved by use of the EHRs telehealth capabilities or other tools for secure HIPAA compatible video conferencing. For this study, we plan to use the Partner Violence Screening (PVS) for universal screening. The PVS is a three-item brief instrument assessing past year IPV and has been used in emergency departments and primary care practices. The instrument demonstrates good sensitivity and specificity as well as established predictive validity.

The instrument will be delivered through a computerized questionnaire. The approach for administering the questionnaire is novel, developed at MUSC, and allows the examination room computer to be locked for clinical care purposes and taken over by the patient, for use in questionnaires and other tasks. Patients demographic profile and the clinic by which they are received care determines whether the patient is presented with the questionnaire. In this

case, all women ages 18-49 in primary care clinics at MUSC will have the IPV screening questionnaire added to their questionnaire queue for completion during the visit. After the patient is roomed by a medical assistant and vital signs recorded, the medical assistant locks the screen of the EHR and then brings up the questionnaire program. The patient then completes the questionnaire. Alternatively, the patient can use a tablet or other computing device if clinics preferred this workflow. The questionnaire is automatically scored within the EHR after completion and when the provider resumes use of the EHR, if the patient screens positive for IPV, a “blocking” (no further actions allowed) pop-up alert shows the results of the questionnaire. If the patient does not screen positive for IPV, provider workflow within the EHR continues as normal.

The provider will then be able to click on a link in the alert to be taken to a secure portion of the electronic health record for the patient. A template will open to assist the provider diagnosis of IPV and with evaluation of the risk levels posed by the current situation of the patient. The provider will then either refer the patient for immediate counseling via telehealth services by the National IPV Hotline or to MUSC social services for immediate in person consultation, if risks to the patient are high for subsequent violence.

The provider will complete the note documenting care in the secure department and other activities required to bill for counseling services for IPV. The patient will be enrolled in a high IPV risk registry for further follow up by the provider and case managers. An IPV diagnosis will not be added to the EHRs problem list to prevent accidental disclosure but alerts will notify providers of information available in a confidential department. Selected providers (primary care, psychiatry, ED, etc.) will be able to “break the glass” and access secure notes during subsequent visits.

All software will be developed using user centered design methods. Input from users will guide successive iterations of modifications of the EHR system to achieve a result that is maximally functional. All software reflects modifications to existing capabilities of the EHR system at MUSC, except for the ability to switch automatically between routine clinic records and the “secure” area of documentation of IPV care (secure areas exist—just not automated switching of context.)

To evaluate this new EHR functionality, in Aim 2, we propose to conduct a stepped wedge cluster randomized trial of deployment of this intervention in primary care clinics. The stepped wedge approach is a pragmatic design that is maximally acceptable to implement and evaluate the intervention (IPV screening, diagnosis and referral). Through this approach all participating practices will be exposed to the intervention, and the random allocation of start times is considered a fair approach to use to account for the fact that it is not practical to implement the intervention in all practices at once. It is also a statistically powerful approach that allows proper evaluation of the impact of the intervention as each clinic can serve as its own contemporaneous control. In our model, all clinics will receive the intervention after a baseline observation period which varies between clinics as randomized. Over time, clinics are randomized to receive the EHR screening intervention to systematically screen all women in the practice.

MUSC Health Primary Care provides services via 28 practices spread across three counties in South Carolina that employ over 40 Physicians (Family Physicians and General Internists) and 10 Nurse Practitioners and Physician Assistants. MUSC Women’s Health (WH) provides care through five practices that employ over 20 gynecologists and nurse midwifes. The two WH sites with the largest primary care practice will be used. Although there are differences in the sites regarding staffing and size, all sites use the same EHR and have access to practice reports with data extracted from the EHR that evaluate the quality of care provided. Most sites have received recognition as a Patient Centered Medical Home (PCMH) through the Nation Committee for Quality Assurance (NCQA), and currently use EHR prompts to provide guideline-based screening, although no prompts are currently available for IPV screening

The two main comparators in the current study are current best practices for screening for IPV (control) and EHR supported IPV screening, diagnosis, counseling, referral, and billing (intervention). Our control condition was selected based on current guidelines for IPV screening and follow-up care. Our intervention condition was selected, consistent with findings from the NCKP approach (Miller, McCaw et al. 2015), given its evidence-based potential to leverage EHRs as an additive support for the implementation of the best practices in IPV care that constitute our control condition. The stepped wedge cluster randomized design of the study will allow us to isolate the causal effects of EHR support on outcomes of interest (e.g., IPV disclosure, referral, practitioner and patient satisfaction) by allowing each cluster of clinics to provide observations for both the control and the intervention conditions. In addition, this study design will reduce potential for bias introduced by controlling for contamination effects across clinic staff in a given setting.

Working with stakeholder representatives, we will develop a formal study protocol specifying the purpose for which data will be collected (comparative effectiveness as fully described in the evaluation plan), data sources and linkage plans, data feasibility and quality, and measures of effect. During Project Year 1 , we will develop a formal study protocol that includes the following core elements: (1) Design for Epic modifications, including screening questionnaires, diagnostic interview support, referral order set and smart phrases, and “hidden” department documentation of counseling and billing; (2) Specification of workflow and standard operating procedures (SOPs) for screening, diagnosis and referral in practical clinic settings; (3) Specification of workflow and SOPs for hotline staff; (4) Manualized training materials for clinic physicians and staff regarding use of Epic software and screening tools, as well as manualized Helpline training materials regarding workflow, software documentation tool modifications, and operations modifications; (5) Data extraction protocol specifying fields in Epic to be extracted and mapping to Research Data Warehouse, an SOP

for extraction and insertion of patient data from interviews into REDCap, an SOP for extraction from REDCap and integration with EHR data for analytic file production, and a data dictionary; and (6) Data analysis plan.

To prepare clinics for participation, we will work with the Primary Care Service Line Director, clinical leaders in Internal Medicine, Gynecology and Family Medicine and staff to implement the study. Prior to roll out in each clinic, we will develop and train relevant clinic staff and providers using the following tools and approaches: 1. Manual for clinic operations of the system 2. Online training for helpline 3. Pre-intervention in-service, online education, and certification for clinic staff and providers. Online training will be integrated with MUSC's MyQuest staff education system. Completion will be mandatory but incentivized ($5 Starbucks card) and rates monitored. During the initial week of the roll out, Epic research support personnel will be available in the clinics to help providers learn to use software modifications and to answer questions. Provider support for in-person individual IPV-related training and case consultations will be provided by two experts in IPV training and management through-out the implementation and operation of the study.

This study is a clinic-level stepped wedge randomized trial. The major sources of potential bias in data collection within a cluster-RCT design include 1) inappropriate selection of sites, and 2) inadequate data collection once subjects are enrolled within a site (Campbell 2014). To address issue #1, clinics will be stratified and randomized in blocks of four. The randomization will be conducted by the study biostatistician and will be stratified by two factors: Total Number of Clinic Encounters by main age risk group (Factor 1) and Type of Clinic (FM, IM, or GYN (Factor 2). First the clinics will be ordered by Factor 1 and divided into 4 subgroups (blocks), with each including 4 clinics. Clinics in each block will be further ordered by Factor 2, and assigned to active intervention start as first, second or third group, making sure that the groups will be balanced in terms of both Factor 1 and Factor 2. To address issue #2, EHRs of all participating clinics will be analyzed every two weeks for screening failures, refusals and data completion rates. De-identified aggregate data completion rates will be provided to the individual clinics through the site champion on a biweekly basis. In person meetings with the site staff to discuss data collection rates for the previous month and cumulative-to-date will be reviewed for each clinic and discussed with staff as a whole as a measure of quality improvement. This approach will allow rapid identification of potential barriers to screening, referral and data collection and rapid and relevant quality improvement interventions to address barriers. Distributions of baseline characteristics for clinics and patients will be compared between the control condition and the active screening condition to assess effectiveness of the randomization, and statistical or clinical differences will be adjusted for in sensitivity analyses. All analyses for treatment group comparisons will use an intention-to-treat approach. Primary and secondary outcomes are described elsewhere.

Certain patient subgroups are of particular interest because they have been reported to be of greater risk for and it is not known if counselling is more or less acceptable or effective for these groups (Black, Basile et al. 2010); Black and colleagues report that relative lifetime risk of IPV is 1.26 for black women and 1.09 for Hispanic women when compared to whites. Further, risk of IPV is highest in women under age 25. We will have adequate enrollment of black women and young women to have statistical power to conduct subgroup analyses of patients in these high-risk subgroups to examine important characteristics of the intervention. We will not be able to examine difference for Hispanic, Asian, or Native American women because of low representation of these groups in SC. We will also explore difference in women living in rural communities and in low income geographic areas by using patients' home address to geocode and construct such measures for the study population. Differences in rates of screening refusal, rates of positive response on the screening questions, variations in types of IPV responses, acceptance of counselling and use of the Hotline will be examined by sub-groups defined by age [18-25 vs. 26-49 years; black vs. non-black race; rural vs non- rural census tracts; and low-income census tracts (to be defined)].

Assessments of the impact of interventions will be performed extraction of data from the EHR system documenting IPV screening and care. The patient-level data for this study are linked within the EHR and REDCap (used to capture data for follow up interviews). Data on clinic characteristics will be merged onto the analytical data set using the clinic identifying number and manually checked for validity. A research data honest broker, a service supported by MUSC's CTSA, will use this to create anonymized files free of HIPAA identifiers (MRN converted to a study ID, dates will be shifted to relative dates, birthdate to age, etc. Social demographic factors will be obtained by geolinkage. An unduplicated list of patient census tract codes will be used to extract data from public use files on urban- rural status, indicators of poverty (e. g. percent of population below federal poverty limits, median income) will be constructed. These variables will then be merged onto the analytical data sets after the data has been extracted from the EHR. Standard data quality assurance approaches will be used to assure valid assignments. Missing values will be identified and treated as discussed in the evaluation plan.

We will also measure provider and clinic staff satisfaction with Epic modifications and the counseling and referral process. Surveys will be conducted by email and EHR messaging tools.

Patient centered outcomes will be collected in patients who agree to be contacted by telephone by the study team (during their clinical visit as recorded at the end of the screening questionnaire for IPV risk) and who subsequently provider oral consent during the phone call. Responses to survey questions administered during the interviews will be recorded in REDCap software and merged with other study data.

4.2.b. Primary Purpose | Screening

4.2.c. Interventions

Type	Name	Description
Other	EHR modifications to screen from Intimate Partner Violence	Modifications a commercial EHR to support screening for IPV using a validated questionnaire, alerts in the EHR to force provider actions to address IPV risks identified, templates for assessment of risk and counseling by the provider, tools within the EHR for a secure handoff to IPV counseling by a national telephone hotline, and modification to the EHR to keep documentation secure and prevent accidental disclosure.

4.2.d. Study Phase — Phase 3

Is this an NIH-defined Phase III Clinical Trial? ● Yes ❍ No

4.2.e. Intervention Model — Sequential

4.2.f. Masking ❍ Yes ● No

❏ Participant ❏ Care Provider ❏ Investigator ❏ Outcomes Assessor

4.2.g. Allocation — Randomized

4.3. Outcome Measures

Type	Name	Time Frame	Brief Description
Primary	Rate of referral to either National Domestic Violence Hotline or social work/security after completion of screening	During the visit	The rate at which patients who screen positive are referred for counseling services (Numerator: Patients referred for coundseling by hotline or social services. Denominator: Patients screening positive for IPV.)
Secondary	Proportion of patients screened	6 months	Rate of screening of eligible primary care patients for IPV (Numerator: all unique patients screened for IPV. Denominator: unique patients meeting study eligibility criteria seen.)
Secondary	Impact on clinic level RVU's of screening	6 months	Total number of RVUs billed by physicians/NP's/ PA in a clinic for a 6 month period
Other	Patient satisfaction with screening	3 months	Patient satisfaction with screening process on a five point Likert scale.
Other	Provider satisfaction with Epic modifications	6 months	Provider satisfaction with Epic modifications and IPV screening workflow (Provider satisfaction with IPV screening and referral process on a five point Likert scale.)

4.4. Statistical Design and Power — 4_4_Statistical_design_and_power1036155727.pdf

4.5. Subject Participation Duration — 90 days

4.6. Will the study use an FDA-regulated intervention? ❍ Yes ● No

4.6.a. If yes, describe the availability of Investigational Product (IP) and Investigational New Drug (IND)/ Investigational Device Exemption (IDE) status

4.7. Dissemination Plan — 4_7_Dissemination_Plan1036155725.pdf

4.4 Statistical Design and Power:

The study main outcome is the rate of successful completion of IPV screening in primary care patients that is followed by the completion of provider assessment, diagnosis and referral in patients who are identified as high risk. Secondary outcomes include: 1) rates use of the National Hotline by patients at risk; and 2) differential rates of IPV screening and counselling for patient subgroups defined by race, age, income and rural location. We will use a generalized linear mixed model with a logit link controlling for block set (see Randomization) and a fixed effect for each step, as discussed by Hussey and Hughes (2007)], to compare active screening accounting for the cluster design with a random clinic effect. Primary analyses will also adjust for patient characteristics (age, race) since these have been associated with risk of IPV. Models will be estimated with interaction terms to assess subgroup effects for specific patients, and to examine the effect of practices' characteristics on the heterogeneity of the estimates. The intraclass correlation coefficients will also be estimated and reported for screening and counselling to assess the assumptions made for the sample size analyses so that this information will be available to future investigations using similar designs and outcomes. Analysis for the secondary aims will be largely description. The proportion and confidence interval will be estimated using generalized linear models adjusting for clustering. In addition, we will perform sensitivity analyses to assess the effects of early vs. late randomization, potential "learning" effects that may occur as the screening becomes better integrated in practice routines, the effect of the practices' presence of a IPV champion prior to the study, and the effect for practice- specific screening refusal and rate of counselling acceptance on the overall estimate. To address missing data issues, baseline characteristics, such as age, race, and zip code, will be compared between patients with positive and negative screening responses and by referral for counselling to those with missing data by intervention group in order to assess potential biases that may exist in the complete analysis (IOM, 2010). Sensitivity analyses for the primary and secondary outcomes will be conducted using several methods, which have different missing data assumptions: 1) Complete case analyses which assumes missing data are completely at random; 2) Multiple imputation using M=10 imputations, which assumes missing at random; and 3) Assigning contrasting age group, race and income category classifications for missing values differentially by intervention group, which aligns with non-ignorable missingness (the data missingness is nonrandom and related to the actual values.)

Power Calculations:

This study will involve a total of n=28 clinics which will provide two years of data on approximately 68,000 patient visits. Of these, we expect 34,000 to occur during the non-screening steps and 34,000 visits during the intervention period (i.e. Aim 2). The table below summarizes data used in power calculations.

1. Estimated number of potentially eligible study participants: *The number of study participants were estimated based on the number of unduplicated females seen in the 28 primary-care and OB practices during 12 months from 10/1/2015 through 9/302016.*	71,400
2. Total number of study participants expected to be screened: *We expect* ***33,915*** *to receive current practice treatment and* ***33,915*** *to be offered IPV screening.*	67,830
3. We *expect a 95% screening acceptance rate based on reports on acceptability in the literature. The US rate of IPV during 12 months reported in the most recent survey is 5.9%. The US-South Carolina RR of lifetime IPV is 1.17. Thus, we expect that 6.9% of screened women will have experience IPV during the last year and that at least 80% will provide a positive response to our screening instrument. Thus a 5.5% IPV rate is expected during the active intervention times.*	32,219
4. Target sample size: *We expect that a minimum of 75% of women who screen positive will accept counselling in the office and that 50% will contact the Hotline during the active phase of the study*	1,329<br>and 886
5. If applicable, total number of practices or centers that will enroll participants: *14 Family Medicine, 12 Internal Medicine Primary Care and 2 women's health clinics*	28
6. Projected month first control group data extract (month after project initiation):	9 months
7. Projected month last active participant enrolled (month after project initiation):	33 months
8. Projected rate of enrollment (anticipated number enrolled 33,912 over the 12 -month active enrollment period): *These are the expected number of women screened per month*	2,423

9. Estimated percentage of participant dropout: *We do not expect drop outs because the study is a one-time screening. However, we do expect refusals and for subjects who decline counselling which will be considered in estimates of effectiveness.*	NA

Hypothesized Effect Size: Studies report the ability of screening to increase patient referral to services from a 2.5 percentage point to an 8.0 percentage point increase based on current referral rates between 0% and 3.6% (O'Doherty et al, 2014). Based on an analysis of our archival data, we expect that our current referral rate for IPV for women age 18-49 seen in our clinics is no more than 0.14% and that this rate will increase to 3.9% with screening.

Based on our current practice visit patterns for unduplicated visits by women between age 18 and 49 years, we will have approximately 34,000 eligible visits during the non-screening steps and 34,000 during the screening steps within the n=28 participating clinics. We have assumed an annual IPV prevalence of 5.5% within the preceding year, based on rates reported by the CDC for SC (Black et al., 2011), a screening completion rate of 95%, a 75% counselling rate for women who screen positive, and a 50% referral rate to the Hotline. With about 600 relevant patient visits over 6 months per clinic, 8-12 clinics per wedge, and 3 wedges, we estimate that we will have 97% power to detect an increase in screening (i.e. 0.14% vs. 5.5%), and 89% power to detect a difference in the rate of referral for counseling and/or calls to the Domestic Violence Hotline (i.e. 0.14% vs. 3.9%).

While we may be somewhat overpowered for our primary analysis, these numbers of clinics and patients will also allow us to make very precise estimates of the treatment effects within relevant patient subgroups (e.g. whites vs. blacks, urban vs. rural) and to determine whether there are any differential impacts of the intervention on those subgroups. These power calculations, conducted using PASS15 (NCSS, Kayesville, UT) and verified using the 'steppedwedge' function in STATA (StataCorp, College Station, TX), assumed an intraclass correlation coefficient of 0.05 (to account for a moderate level of clustering of patients within clinics), along with an alpha of 0.05 and two-sided testing.

4.7 Dissemination Plan

The use of a nationally widespread EHR system (EPIC) and the integration of population health and billing tools as well as leveraging the counseling and referral resources of the National Domestic Violence Hotline (to overcome potential regional IPV resource barriers) enhance ease of dissemination of this project. IPV screening in primary healthcare settings has been endorsed by numerous professional associations including the IOM , USPSTF the World Health Organization , American College of Obstetricians and Gynecologists , American Academy of Family Physicians, American Nurses Association, and the American Academy of Pediatrics. We will engage these organizations to disseminate results. To aid in future dissemination, we would study how to adapt ongoing efforts to other EHR platforms, including Cerner and Meditech, focusing on developing standards such as the Fast Healthcare Interoperability Resource protocol.

To support in early adoption of tools, all software programming and training materials will be available on request to any Epic installation. Working with Epic, will we will make a toolkit available on the Epic User Web (see letter) with including training materials within 18 months of the start of the proposal. In addition to presentations at the usual academic meetings, we will submit proposals for Epic Expert (XGM) and User Group (UGM) meetings (annual meetings that host thousands of clinicians and IT experts). If successful, we will work with Epic to make this installation part of its base system. To help adopters implement and sustain implementations, we will have a monthly conference call, to provide ongoing support for implementation. MUSC is also launching an external fee-for-service Epic Research consultation service. This team will be able to provide direct support for implementation of the software and programs developed.

We also will work regionally on direct dissemination through Health Sciences South Carolina, a regional learning health system collaborative, to encourage its members that use Epic systems (Anmed Health, Self-Regional Health, and Spartanburg Health systems) to implement this screening program. Health Sciences also works with Duke, UNC Chapel Hill and Wake Forrest universities through the Carolinas Collaborative (CC), which has a shared data system spanning these universities and pilot funding mechanisms for cross institutional research to support dissemination efforts. All of the CC sites use the Epic EHR and could be part of a future scaling study.We will also work with Dr. Stern to take innovations to the North Carolina Coalition against Domestic Violence, which is working on EHR implementations of IPV screening for smaller hospitals and Dr. Brigid McCaw to bring refinements of the NCKP program developed herein back to NCKP and to other Kaiser Permanente regions.

Section 5 - Other Clinical Trial-related Attachments (Study 1)

5.1. Other Clinical Trial-related Attachments

Delayed Onset Studies

Delayed Onset Study#	Study Title	Anticipated Clinical Trial?	Justification
The form does not have any delayed onset studies			

Leadership Plan

Governance and organizational structure of the leadership team and the research project

The research team will be led by co-PIs Leslie Lenert, MS, MD, FACMI, and Alyssa Rheingold, PhD. Dr. Lenert is Professor of Internal Medicine, Chief Research Information Officer and the Director of the Biomedical Informatics Center at the Medical University of South Carolina (MUSC). He will serve as contact PI for this project. As a health systems engineer devoted to creating advanced technical processes that address critical problems in population health through information technology, he has expertise in use of electronic medical record systems in guiding screening approaches within medical workflow. Dr. Rheingold is a Clinical Psychologist and Professor in the National Crime Victims Research and Treatment Center (NCVC) within the Department of Psychiatry and Behavioral Sciences at MUSC. Dr. Rheingold is also the Director of Clinical Operations of the NCVC. She has worked clinical for the past 16 years in the area of victimization, with a specialization in interpersonal violence victims. Her research areas focus better understanding the impact of victimization and trauma related to health outcomes and to evaluate prevention, early intervention, and treatment strategies. Dr. Rheingold has significant experience in coordinating randomized controlled studies, both multi- site and community based and is an expert within the interpersonal violence field. Drs. Lenert and Rheingold are both faculty in the College of Medicine at MUSC, and have been collaborating for the past year on this proposal. In their roles as Co-PIs, Drs. Lenert and Rheingold will be jointly responsible for carrying out the study aims, fiscal and administrative management including maintaining communication, IRB approval and coordination of administrative reports. Drs. Lenert and Rheingold will also share responsibility in disseminating study findings through published manuscripts, conference presentations, and open source algorithms and web applications. The research team will also include Kathleen Brady, MD, Kit Simpson, PhD, Paul Nietert, PhD, Jenna Mccauley, PhD, Vanessa Diaz, MD and Angela Moreland, PhD. All Co-Is have relevant specific expertise that will aid to the overall design, analyses, interpretation, and dissemination of results. All Co-Is will report directly to the PIs. Both PI's will communicate with the study consultants and stakeholders. Financial and administrative aspect will be handled by the administrative staff of the Biomedical Informatics Center at MUSC.

Administrative, technical, scientific, and engagement responsibilities for each PI and the rationale for submitting a dual-PI application

Our proposed dual leadership plan is critical to the success of this project, given that our proposal brings together several unique facets of medical informatics and interpersonal violence screening. Because the two PIs complement each other's technical and leadership expertise, both are essential for a successful completion of the proposed study. Specifically, Dr. Lenert brings expertise in informatics, system design, and outcomes research. Dr. Rheingold's expertise in dynamics of interpersonal violence and community based RCTs are required to achieve best screening tools within Aim 1, implementation approaches to best practice of screening, counseling and referral of IPV with Aim 2, and multisite community based RCT experience for research design.

Drs. Lenert and Rheingold will oversee all aspects of the project. They will be responsible for the implementation of the research plan, ensuring that systems are in place to guarantee institutional compliance with US laws, AHRQ policies and institutional guidelines related to human subjects' protection and data security. Dr. Lenert will serve as contact PI for the project and will coordinate various aspects of the study including supervision of selection of sites and preparation of training materials with other team members. He will be responsible for MUSC institutional IRB approval, communication with AHRQ project officers and staff, completion of annual milestone reports. During the development phase of the study (Aims 1), he will work closely with the informatics team on the creation of the EHR tools. He will take the lead on Aim 2 of the study, working closely with Dr. Simpson and Diaz on the implementation of the stepped wedge randomized cluster design. Dr. Lenert will also lead the examination of surveillance, alerting and summarization of EHR data on rates of referral to counseling services (Aim 3).

Dr. Rheingold will be the subject matter expert, taking the lead on Aim 1 overseeing the development of developing a systematic process for using an EHR in combination with validated IPV tools for screening, counseling, and referral. She will work closely with Dr. Diaz in family medicine on workflow approaches and Drs. Moreland and McCauley on IPV best practices and enhanced practice approaches for adoption. In Aim 2,

as the principal subject matter expert, she will lead the deployment of the screening system in clinics. Further, she will serve as primary point of contact for stakeholder engagement throughout all AIMS of the project to ensure community and patient involvement. She will work closely with Dr. Lenert to assist in Aims 2 and 3 on design protocols, ensuring confidentiality and addressing the unique needs of IPV victims in the resign design and future applicability of intervention approaches. Throughout the study, she will assist Dr. Lenert in administrative responsibilities related to IRB, annual reports, and communication with AHRQ administrative staff.

A publication policy will be established based on the relative scientific contributions of the PIs and key personnel. As, part of this publication policy, both Drs. Lenert and Rheingold will be listed and included as authors on publications and presentations that may result from significant findings.

Intellectual property

Dr. Rheingold and Dr. Lenert will form a Steering Committee comprising the PIs, Co-Investigators, consultants, and patient and community stakeholders that will coordinate project management, research administration, publications, data sharing, and integration of all resources. The Steering Committee will communicate bi-weekly, either by phone, e- mail, or in person, to discuss experimental design, data analysis, all administrative responsibilities, and any changes in the direction of the research projects. Both PIs share their respective research results with one another, co- investigators, and consultant. A publication policy will be established based on the relative scientific contributions of the PIs, key co-investigator, and developers.

The PIs will grant necessary access rights to the pre-existing patents and or the patents potentially generated within the frame of this project for the purpose of this research project to all the other key personnel on a non-exclusive royalty- free basis. Each PI shall take appropriate measures to ensure that he/she can grant these access rights. Right in any pre- existing intellectual property will remain the property of the party that created and/or controls it.

Conflict Resolution

If a potential conflict develops, the PIs shall meet and attempt to resolve the dispute. If they fail to resolve the dispute, the disagreement shall be referred to Dr. Kathleen Brady, MUSC’s VP for Research, and a co-investigator, who encourage compromise but will have the authority to make a binding ruling.

Changes in PI location

If a PI moves to a new institution, attempts will be made to transfer the relevant portion of the grant to the new institution. In the event that a PI cannot carry out his/her duties, a new PI with appropriate expertise (informatics or context) will be recruited as a replacement.

References:

American Academy of Family Physicians. Violence position paper: AAFP policy and advocacy statement. American Academy of Family Physicians. 2005.
American College of Obstetrics and Gynecology. ACOG Committee opinion no. 518: Intimate partner violence. Obstet Gynecol, 2012. 119(2): p. 412-7." PMID: 22270317
American Nurses Association. Position statement on physical violence against women. Washington, DC: American. Nurses Association. 1991.
Bureau of Justice. Statistics, BOJ, Criminal Victimization, 2010. 2011: Washington DC.".
Bakken, S. and C. M. Ruland (2009). "Translating clinical informatics interventions into routine clinical care: how can the RE-AIM framework help?" Journal of the American Medical Informatics Association **16**(6): 889-897. PMCID: PMC3002125
Black, M., K. Basile, M. Breiding, S. Smith, M. Walters and M. Merrick (2010). The National Intimate Partner and Sexual Violence Survey (NISVS): Summary Report. Atlanta, GA: National Center for Injury Prevention and Control, Centers for Disease Control and Prevention, 2011.
Breiding, M. J. (2014). "Prevalence and characteristics of sexual violence, stalking, and intimate partner violence victimization—National Intimate Partner and Sexual Violence Survey, United States, 2011." Morbidity and mortality weekly report. Surveillance summaries (Washington, DC: 2002) **63**(8): 1. PMCID: PMC469245
Brock, K. (2003). "When men murder women: An analysis of 2001 homicide data." Washington, DC: Violence Policy Center: 1-33.
Brown, C. A. and R. J. Lilford (2006). "The stepped wedge trial design: a systematic review." BMC medical research methodology **6**(1): 1. PMCID: PMC1636652
Campbell, J. C. (2002). "Health consequences of intimate partner violence." The Lancet **359**(9314): 1331-1336. PMID: 11965295
Campbell, J. C., D. W. Webster and N. Glass (2009). "The danger assessment validation of a lethality risk assessment instrument for intimate partner femicide." Journal of interpersonal violence **24**(4): 653-674. DOI: 10.1177/0886260508317180
Campbell MJ. Challenges of cluster randomized trials. J Comp Eff Res. 2014, May;3(3):271-81. doi: 10.2217/cer.14.21. Review. PubMed PMID: 24969154.
Centers for Disease Control and Prevention. Defintion of intimate partner violence. 2016. https://www.cdc.gov/violenceprevention/intimatepartnerviolence/definitions.html. accsessed 01/05/2017
Centers for Disease Control and Prevention. Costs of intimate partner violence against women in the United States. 2003, Center for Disease Control and Prevention: Atlanta, GA.
Chapin, J. R., G. Coleman and E. Varner (2011). "Yes we can! Improving medical screening for intimate partner violence through self-efficacy." Journal of Injury and Violence Research **3**(1): 19. PMC3134916/
Conybeare D, Behar E, Solomon A, Newman MG, Borkovec TD. The PTSD Checklist-Civilian Version: reliability, validity, and factor structure in a nonclinical sample. J Clin Psychol. 2012 Jun;68(6):699-713. PMID: 22517497.
Cooper, A., R. Reimann, D. Cronin and C. Noessel (2014). About face: the essentials of interaction design, John Wiley & Sons. de Boinville, M. (2013). "Screening for domestic violence in health care settings." ASPE policy brief.
Derogatis LR. BSI-18: Brief Symptom Inventory 18 - Administration, scoring, and procedures manual. Minneapolis: NCS Pearson; 2000.
Dumas, J. S. and J. Redish (1999). A practical guide to usability testing, Intellect Books.
Epic Systems. "http://www.epic.com/about." Accessed December 6, 2016.".
Feldhaus, K. M., Koziol-McLain, J., Amsbury, H. L., Norton, I. M., Lowenstein, S. R., & Abbott, J. T. (1997). Accuracy of 3 Brief Screening Questions for detecting partner violence in the emergency department. *JAMA, 277*(17), 1357-1361 PMID: 9134940
Hemming, K., T. Haines, P. Chilton, A. Girling and R. Lilford (2015). "The stepped wedge cluster randomised trial: rationale, design, analysis, and reporting." BMJ **350**: h391. PMID: 2566294

Houry D, Feldhaus K, Peery B, et al. A positive domestic violence screen predicts future domestic violence. J Interper Violence. 2004;19(9):955–66. PMID: 15296611
Hussain, N., S. Sprague, K. Madden, F. N. Hussain, B. Pindiprolu and M. Bhandari (2013). "A comparison of the types of screening tool administration methods used for the detection of intimate partner violence a systematic review and meta-analysis." Trauma, Violence, & Abuse: 1524838013515759. PMID: 24343478
Hussey, M.A. and J.P. Hughes, Design and analysis of stepped wedge cluster randomized trials. Contemp Clin Trials, 2007. **28**(2): p. 182-91. PMID: 16829207
IOM, "The Prevention and Treatment of Missing Data in Clinical Trials: National Research Council (US) Panel on Handling Missing Data in Clinical Trials." Washington (DC): National Academies Press (US); 2010. ISBN-13: 978-0-309-15814-5ISBN-10: 0-309-15814-1
Kirst, M., Y. J. Zhang, A. Young, A. Marshall, P. O'Campo and F. Ahmad (2012). "Referral to Health and Social Services for Intimate Partner Violence in Health Care Settings A Realist Scoping Review." Trauma, Violence, & Abuse 13(4): 198-208. PMID: 22899703
Klevens, J, Sadowski L, Kee, R, Trick W, Garcia. "Comparison Comparison of†screening and†referral strategies for exposure to partner violence. Womens Health Issues. 2012 JanFeb; 22(1): e4552. PMID: 21798763
Kottenstette, J. B. and D. Stulberg (2013). "Time to routinely screen for intimate partner violence?" Priority Updates to Research Literature (PURLs).
Krug, S. (2014). Don't make me think revisited: A common sense approach to web and mobile usability . Berkeley, CA: New Riders Press.
Letourneau, E. J., M. Holmes and J. Chasedunn-Roark (1999). "Gynecologic health consequences to victims of interpersonal violence." Women's Health Issues **9**(2): 115-120. PMID: 10189822
McCloskey, L. A., E. Lichter, C. Williams, M. Gerber, E. Wittenberg and M. Ganz (2006). "Assessing intimate partner violence in health care settings leads to women's receipt of interventions and improved health." Public health reports: 435-444. PMID: PMC1525344
McFarlane, J. M., J. Y. Groff, J. A. O'Brien and K. Watson (2006). "Secondary prevention of intimate partner violence: a randomized controlled trial." Nursing research **55**(1): 52-61. PMID: 1643992
Miller, E., B. McCaw, B. L. Humphreys and C. Mitchell (2015). "Integrating intimate partner violence assessment and intervention into healthcare in the United States: A systems approach." Journal of Women's Health **24**(1): 92-99. PMID: 25606823
Mills TJ, Avegno J, Haydel MJ. Male victims of partner violence: prevalence and accuracy of screening tools. J Emerg Med. 2006;19(2):117–24. PMID: 17046494
Moyer, V. A. (2013). "Primary care interventions to prevent child maltreatment: US preventive services task force recommendation statement." Annals of internal medicine **159**(4): 289-295. PMID: 23752681
Nelson, H. D., C. Bougatsos and I. Blazina (2012). "Screening women for intimate partner violence: a systematic review to update the US Preventive Services Task Force recommendation." Annals of internal medicine **156**(11): 796-808. PMID: 22565034
Nielsen, J. (1994). Usability inspection methods. Conference companion on Human factors in computing systems, Boston MA, USA. ACM. 1994. DOI: 10.1145/259963.26053
O'Doherty, L. J., A. Taft, K. Hegarty, J. Ramsay, L. L. Davidson and G. Feder (2014). "Screening women for intimate partner violence in healthcare settings: abridged Cochrane systematic review and meta-analysis."
Oehme, K. and N. Stern (2014). "Case for Mandatory Training on Screening for Domestic Violence in the Wake of the Affordable Care Act, The." U. Pa. JL & Soc. Change **17**: 1.
Papers on Preventive Interventions. Forum on Global Violence Prevention; Board on Global Health; Institute of Medicine. Preventing Violence Against Women and Children: Workshop Summary. Washington (DC): National Academies Press (US); 2011 Sep 12. (Chapter) 8, Papers on Preventive Interventions. Available from: https://www.ncbi.nlm.nih.gov/books/NBK236967/
Rhodes, K. V., D. S. Lauderdale, T. He, D. S. Howes and W. Levinson (2002). "“Between me and the computer”: increased detection of intimate partner violence using a computer questionnaire." Annals of emergency medicine **40**(5): 476-484. PMID: 1239979

Robling, M. R., D. K. Ingledew, G. Greene, A. Sayers, C. Shaw, L. Sander, I. T. Russell, J. G. Williams and K. Hood (2010). "Applying an extended theoretical framework for data collection mode to health services research." BMC health services research **10**(1): 1. PMID: 20576131

Rosen, C.S., Henson, B.R., Finney, J.W. & Moos, R.H. (2000). Consistency of self-administered and interview-based Addiction Severity Index composite scores. *Addiction, 95(3),* 419-425. PMID: 10795362

Rosson, M. B. and J. M. Carroll (2002). Usability engineering: scenario-based development of human-computer interaction, Morgan Kaufmann.

Silverman, M. E. and H. Loudon (2010). "Antenatal reports of pre-pregnancy abuse is associated with symptoms of depression in the postpartum period." Archives of women's mental health **13**(5): 411-415. PMID: 2038694

Sullivan CM, Cain D. Ethical and safety considerations when obtaining information from or about battered women for research purposes. J Interpers Violence. 2004 May;19(5):603-18. PMID: 15104864.

Taylor B, Limowski A. Research prioritization topic brief 1: Screening for intimate partner violence. Patient Centered." US_Preventative_Services_Task_Force, Screening for Family and Intimate Partner Violence and Abuse of Elderly and Vulnerable Adults., H.a.H. Services, Editor. 2013: Rockville, MD.".

Walters, M. L., J. Chen and M. J. Breiding (2013). "The National Intimate Partner and Sexual Violence Survey (NISVS): 2010 findings on victimization by sexual orientation." Atlanta, GA: National Center for Injury Prevention and Control, Centers for Disease Control and Prevention **648**(73): 6.

Conybeare D, Behar E, Solomon A, Newman MG, Borkovec TD. The PTSD Checklist-Civilian Version: reliability, validity, and factor structure in a nonclinical sample. J Clin Psychol. 2012 Jun;68(6):699-713. doi: 10.1002/jclp.21845. Epub 2012 Apr 19. PMID: 22517497.

World Health Organization. WHO multi-country study on women's health and domestic violence against women: summary report of initial results on prevalence, health outcomes and women's responses. 2005.

Wingood, G. M., R. J. DiClemente, D. H. McCree, K. Harrington and S. L. Davies (2001). "Dating violence and the sexual health of black adolescent females." Pediatrics **107**(5): e72-e72. PMID: 11331722

Young-Wolff, K. C., K. Kotz and B. McCaw (2016). "Transforming the Health Care Response to Intimate Partner Violence: Addressing “Wicked Problems”." Jama **315**(23): 2517-2518. PMID: 27327796

The Permanente Medical Group, Inc.
1950 FRANKLIN STREET
OAKLAND, CALIFORNIA 94612-2998

ANTIOCH
DAVIS
FAIRFIELD
FREMONT
FRESNO
GILROY
HAYWARD
MARTINEZ
MILPITAS
MOUNTAIN VIEW
NAPA
NOVATO
OAKLAND
PARK SHADELANDS
PETALUMA
PLEASANTON
RANCHO CORDOVA
REDWOOD CITY
RICHMOND
ROSEVILLE
SACRAMENTO
SAN FRANCISCO
SAN JOSE
SAN RAFAEL
SANTA CLARA
SANTA ROSA
S. SACRAMENTO
S. SAN FRANCISCO
STOCKTON
VACAVILLE
VALLEJO
WALNUT CREEK

September 6, 2017

Leslie Lenert and Alyssa Rheingold
67 President Street, MSC 861
Medical University of South Carolina
Charleston, South Carolina 29425

Dear Drs. Lenert and Rheingold:

I commend you on your thoughtful approach to better understand intimate partner violence (IPV) screening approaches in outpatient medical settings. I am delighted to serve as a consultant on your AHRQ application titled *EHR-based screening and intervention for intimate partner violence.* This proposal takes what we know about IPV screening and referral practices to the next level by using novel strategies to enhance medical communication, identification, and intervention using evidence-based IPV screening tools and health information technology.

In my position as the Medical Director of the Family Violence Prevention Program for Kaiser Permanente, Northern California Region, I oversee the implementation of a comprehensive, coordinated approach for improving screening, identification, and services for intimate partner violence (IPV) and lead the national efforts of Kaiser Permanente to improve IPV for our 10 million members. I am also past Chair of the National Health Collaborative on Violence and Abuse, and a member of the National Academy of Medicine's Forum on Global Violence Prevention.

IPV unfortunately occurs too often in the United States. Not only does it have a direct impact on the lives of victims and their families, it costs millions in direct costs of medical and mental health care as well as indirect costs related to lost productivity. Healthcare systems offer a unique opportunity to screen and intervene for IPV in order to connect IPV victims to appropriate trauma-informed care .. Using my background in public health and internal medicine, and experience as Medical Director of the Kaiser Permanente Family Violence Prevention Program, I have been involved in a number of projects examining best practices in the health care response to IPV. I look forward to participating as a consultant on your study. I hope to offer input on lessons learned about implementation, dissemination, the use of tools in the electronic health record, performance improvement methodology, in the context of a patient centered approach.

Sincerely,

Brigid McCaw, MD, MPH, MS, FACP
Medical Director
Kaiser Permanente Family Violence Prevention Program
Oakland, California

KAISER PERMANENTE

09906-44 (12-95)

August 29, 2017

Alyssa Rheingold, Ph.D.
National Crime Victims Research and Treatment Center
Medical University of South Carolina
67 President Street, MSC 861 Charleston, S. Carolina 29425

Dr. Rheingold:

As a survivor of domestic violence myself, I know firsthand the impact of violence on physical and psychological health. I was fortunate to have family support and be in position to leave my abuser. Other women are not as fortunate. Since my experience, I have made it a mission to do what I can to help other women in need so that they are not alone. I an excited to serve as a patient co-investigator to represent the voice of those victims and survivors who have not been heard. I am very impressed by the work that has been done at the National Crime Victims Research and Treatment Center. It excites me to know that you are submitting an AHRQ grant application, titled "EHR-based screening and intervention for intimate partner violence"to develop a systematic process for using an electronic medical records system and assess the impact of implementing a screening intervention and referral process.

As you are aware, since my experience, I have worked at My Sister's House Inc., a local domestic violence shelter for the past 28 years (two years as the Director of Public Relations/Volunteers and 26 years as the Executive Director). Here at My Sister's House, my staff and I are witnesses to the physical, emotional and psychological effects of intimate partner violence each day. We understand the difficulty of identifying individuals that are in need of services related to consequences of intimate partner violence, especially due to low rates of disclosure and access to services. In addition, we are well aware of the lack of validated instruments to screen for intimate partner violence within health care settings. Developing a systematic process for assessing intimate partner violence, as well as assessing this process, is critical to identification of those in need of services and effectiveness of this screening process.

Further, each intimate partner violence victim has a particular set of needs, which are not always easy to address with the current systems in place. Thus, it is often difficult to identify which victims should be referred for counseling services, or to implement these procedures. For this reason, as both a survivor and on behalf of all of the staff at My Sister's House, we applaud your efforts to better confront the many challenges of identification, assessment, and referral to services among victims of interpersonal violence.

I understand that intimate partner violence is a common denominator among all socioeconomic backgrounds and is extremely prevalent across South Carolina. The NCVC and MUSC have always demonstrated the ability to connect victims to services and to collaborate with organizations throughout South Carolina to provide and refer victims to services as needed. We are proud to have worked alongside such an admirable, hardworking team of individuals at the NCVC and MUSC, and look forward to continuing our relationship between programs. We offer our support on your application for an AHRQ grant, as we know this will tremendously help survivors.

Best Regards,

Ms. Elmire Raven

Elmire Raven, M.A.

Leslie Lenert and Alyssa Rheingold
67 President Street, MSC 861
Medical University of South Carolina
Charleston, SC 29425

September 1, 2017

Dear Drs. Lenert and Rheingold:

I am honored to serve as a consultant for the Medical University of South Carolina's AHRQ proposal entitled "EHR-based screening and intervention for intimate partner violence." My understanding is this research proposal will look at ways to potentially improve best practice screening approaches for intimate partner violence (IPV) identification and intervention. Better understanding of screening approaches and connecting victims of IPV to professionals trained in working with violence victims is sorely needed. Often providers are unsure how to ask and hesitant to intervene when IPV victims present in clinics. This project would allow for systematic guidance and direction for providers in screening and referring to appropriate care.

I am a national consultant working to inspire action to end gender-based violence. From 2009-2014 I served as the first-ever White House Advisor on Violence Against Women. As a senior advisor to Vice President Biden, I developed new initiative to address sexual violence, reduce domestic violence homicides, and improve workplace violence against women. I was Executive Director of the National Network to End Domestic Violence and recently served as Vice President for Strategic Partnerships at the National Domestic Violence Hotline.

I have participated over the past nine months in your preparation stages of this proposal. We have spoken several times to discuss the design, measurements, and referral coordination with the National Domestic Violence Hotline. I have appreciated your attention to issues raised related to confidentiality of participants who may call the Hotline related to data collection for the study. Your diligence in ensuring the safety of this patient population is significant and important as you continue to develop the design and protocols. Given my expertise as a nationally recognized IPV advocate, I hope to continue to offer perspectives of IPV victims in your study approach and interpreting outcomes.

Please let me know what I can do to further assist in this application process. I look forward to our collaboration on this valued effort. It is research like this that is sorely needed to address the public health impact of violence against women.

Sincerely,

lynn rosenthal

Lynn Rosenthal
Consultant

September 5, 2017

Leslie Lenert, Ph.D. and Alyssa Rheingold, Ph.D.
Medical University of South Carolina
67 President Street, MSC 861 Charleston, S. Carolina 29425

Drs. Lenert and Rheingold:

I look forward to serving as a Patient Co-Investigator on The Medical University of South Carolina's AHRQ grant application, titled *EHR-based screening and intervention for intimate partner violence*. I understand that this proposal will examine the use of electronic medical record systems in helping providers screen, identify, and refer victims of domestic violence to a national hotline.

I am honored to serve as a Patient Co-Investigator, representing the views of domestic violence survivors in the development and design of this project. My personal experience with intimate partner violence impacted my life in so many different ways. I struggled but persevered during the difficult times when I left my ex-husband. It was an experience that I do not wish any individual and their family should endure. It impacted my and my son's life forever. However, from those dark years, I have grown into a confident woman who has a strong voice for seeking advocacy for others in need. I currently am involved with a local non-profit organization, Liza's Lifeline of South Carolina, Inc, whose mission is to help domestic violence victims and their families through financial assistance, education and social change.

I hope that my experience and perspective as a survivor provides insight and direction throughout this research study. I have appreciated your request for my input on the proposal process and feel that I will be an integral member of the team once the project is up and running.

Thank you for your efforts in making a difference in the lives of violence victims and survivors.

Best,

Leslie Sommerdyke

HEALTH SCIENCES SOUTH CAROLINA
A Collaborative To Advance Health Sciences

Office of the President

September 13, 2017

Dr. Leslie Lenert
Chief Research Information Officer and Director
Center for Biomedical Informatics
South Carolina Translational Research Institute
Medical University of South Carolina
Charleston, South Carolina 29425

Dear Dr. Lenert:

I would like to express HSSC's enthusiastic support for your proposal entitled "EHR-based screening and intervention for intimate partner violence." This project addresses a critical issue for our community, with Intimate Partner Violence (IPV) being unfortunately common in South Carolina. It uses an advanced approach to screen, diagnose and refer patients for IPV treatment within Epic that builds of tools created by Northern California Kaiser Permanente, extending these for work within fee for service environments.

HSSC operates the Carolina eHealth Alliance (CeHA) health information exchange for its members. This HIE would be a logical place to disseminate innovations moving from Epic to Cerner and MediTech platforms. We would be delighted to work with you to help extend this project's work across CeHA. HSSC is a statewide organization, and its members include three institutions (Self, AnMed and Spartanburg Health Systems) that use Epic EHRs. We look forward to working with you to extend this work statewide, if warranted, to improve the health of all South Carolinians.

Congratulations on development of such an innovative concept and proposal and I wish the best of luck with your application.

Best regards,

Kenneth R. Deans, Jr.

Kenneth R. Deans, Jr., MBA
President & Chief Executive Officer

1320 Main Street, Suite 625, Columbia, SC 29201 • Ph 803-576-5902 • Fx 803-576-5577

MUSC
MEDICAL UNIVERSITY of SOUTH CAROLINA

NATIONAL CRIME VICTIMS RESEARCH & TREATMENT CENTER
Department of Psychiatry and Behavioral Sciences
67 President Street ◆ 2nd Fl. IOP South Bldg.
MSC 861
Charleston, SC 29425-8610
Tel 843 792 2945
Fax 843 792 3388
www.musc.edu/ncvc

September 1, 2017

Dear Les and Alyssa:

I am pleased to accept your invitation to serve as part of the Advisory Group for your proposed project to AHRQ. This project will develop a systematic process for using an electronic medical health records system (EHR) in combination with validated instruments to screen patients in primary care settings for intimate partner violence; alert providers of potential problems; and assist providers with appropriate patient referrals. Results of this project will move the field forward to meet the growing challenges in working with victims of intimate partner violence.

I enthusiastically support your application and look forward to participating in the quarterly Advisory Board meetings. I understand my responsibilities will be to review the progress and safety of all project components and procedures, as well as to share perspectives on data interpretation. Throughout the project, I will help to ensure patient-centeredness and feasibility in working with victims of intimate partner violence.

As you know, I have a strong interest in these activities, serving as a Professor and Director of Adult Services at the National Crime Victims Research and Treatment Center (NCVC) at the Medical University of South Carolina (MUSC). The focus of my work is on the effects of victimization and psychological trauma. I have served on numerous National Advisory Committees (e.g., the National Academy of Science's Institute of Medicine; the Joint NIMH/DOD/DOJ Mental Health and Mass Violence Consensus Committee; the Secretary of Defense Advisory Committee on Women; the International Association of Chiefs of Police). During 2015-16, I also served on S.C. Governor Nikki Haley's Statewide Taskforce on Domestic Violence. I have received numerous awards for my work with crime victims including the U.S. Department of Justice/OVC Award for Outstanding Service on Behalf of Victims of Crime presented by President Clinton and Attorney General Reno in 1996.

Because of the breadth of this work, this team is extraordinarily well-suited to conduct this important project. I look forward to working with you, your partners, and the other Advisory Board members should you be awarded this grant.

Sincerely,

Connie L. Best, Ph.D.

Connie L. Best, Ph.D.
Professor

"An equal opportunity employer, promoting workplace diversity."

MUSC Health
Medical University of South Carolina

Acute, Critical & Trauma Care ICCE
169 Ashley Avenue
744 North Tower
MSC 351
Charleston, SC 29425-3470

Alyssa Rheingold, Ph.D.
National Crime Victims Research and Treatment Center
Medical University of South Carolina
67 President Street, MSC 861
Charleston, SC 29425

5 September 2017

Dear Drs. Lenert and Rheingold:

As you are aware I am the Medical University of South Carolina's FNE Program Coordinator. I have collaborated and coordinated care for victims of interpersonal violence with the National Crime Victims Center for the past five years. I am very excited to serve on the Advisory Board for this AHRQ grant submission to examine the use of the electronic medical record system in better screening and follow through care for intimate partner violence. Information from this study not only will improve the care to victims within MUSC but also be useful as a standard model to optimize the use of electronic health records in prompting evidence based screening and intervention for intimate partner violence.

The MUSC Forensic Nurse Examiner (FNE) Program was formally developed in 2010 to provide sexual assault medical forensic evaluations 24 hours a day by specially trained nurses. Of those sexual assault victims we serve, roughly 15% self-disclose the sexual assault was related to domestic violence, a known high risk factor for potential increased harm. Our Program works on safety planning with these victims, but also is active in education across the University for other healthcare professionals on intimate partner screening, intervention, and local resources. As MUSC's FNE Coordinator, I have been working closely with MUSC administration to create more standardized approaches and apply best practice to screening and intervention for intimate partner violence across MUSC outpatient and inpatient clinics. I am the Co-Chair of MUSC's Domestic Violence Awareness Committee and helped create the online training course for MUSC staff and faculty on working with domestic violence victims.

I am thrilled to work on this AHRQ grant as part of a multidisciplinary Advisory Board to assist in providing further support and care for victims of intimate partner violence as well as better educate health care professionals in best practice for screening and intervention. I am committed to attending quarterly meetings to review progress and identify safety concerns, as well as to review any information pertinent to the project.

I look forward to being a part of this critical initiative to improve services for victims of intimate partner violence both at MUSC and hopefully with our findings nationally.

Sincerely,

Kathy Gill-Hopple

Kathleen Gill-Hopple, RN, PhD, SANE-A SANE-P

FNE Program Coordinator
Medical University of South Carolina

"An equal opportunity employer, promoting workplace diversity."

MUSC
DEPARTMENT of PSYCHIATRY
& BEHAVIORAL SCIENCES

Brain Stimulation Service

67 President Street
PO Box 250861
Charleston, SC 29425
Tel: 843-792-5716
Fax: 843-792-5702

http://academicdepartments.musc.edu/psychiatry/research/bsl

Alyssa Rheingold, Ph.D.
National Crime Victims Research and Treatment Center
Medical University of South Carolina
67 President Street, MSC 861
Charleston, SC 29425

August 21, 20167

Dear Alyssa and Leslie,

I am pleased to learn that you are applying for an AHRQ grant to develop a systematic process for utilizing the EPIC electronic health records system in primary care settings to screen patients for intimate partner violence, then to alert providers of concerns and assist in referring victims to appropriate services. I warmly support you're application, and would be honored to serve on the Advisory Board for this significant and unique project.

As an Associate Professor in the Brain Stimulation Division, as well as the Informatics Medical Director, the aims of this project are very central to my interests and expertise. Specifically, I am an academic clinician and researcher in the Department of Psychiatry. As the Informatics Medical Director, I enhance healthcare delivery through effective information technology that rekindles the doctor-patient relationship, while coordinating care across complex medical systems.

I strongly support your application and look forward to participating in the quarterly Advisory Board meetings, as well as assisting throughout the duration of the project. I understand that my responsibilities will be to ensure the feasibility and safety of the intervention and procedures, as well as to promote patient-centeredness. I agree to attend all Advisory Board meetings, review the progress of the project aims, identify safety concerns, and assist in interpreting the data collected.

In sum, I am very excited to be a part of this novel project that will surely contribute to improving the identification, assessment, and referral of victims of intimate partner violence. I look forward to serving as an Advisory Board member and collaborating with this qualified team.

Sincerely,

Baron Short, MD, MSCR
Associate Professor of Psychiatry and Medicine
Brain Stimulation Service Medical Director
Information Technology Medical Director

"An equal opportunity employer, promoting workplace diversity."
Printed on 100% Post Consumer Recycled Paper

Medical University of South Carolina
Institute of Psychiatry
#67 President Street, MSC 861
Charleston, SC 29425
email: shorteb@musc.edu

"An equal opportunity employer, promoting workplace diversity."
Printed on 100% Post Consumer Recycled Paper

Leslie Lenert and Alyssa Rheingold
67 President Street, MSC 861
Medical University of South Carolina
Charleston, SC 29425

September 1, 2017

Dear Drs. Lenert and Rheingold:

I fully support your AHRQ proposal to examine best informatics practices for intimate partner violence (IPV) screening within primary care setting. I believe that the aim of this study, to develop systematic process for using electronic health records systems for IPV screening, is the next step in better serving IPV victims within healthcare systems. A number of initiatives in developing best practice for IPV screening have been developed, however this proposal builds on the use of informatics practices in optimizing such best practice to determine whether such approaches improves screening and directs providers to consistent appropriate referral streams.

I truly support this proposal and look forward to participating in the quarterly Advisory Board meetings. I understand my responsibilities will be to offer guidance in best practice for IPV screening and intervention within healthcare settings and offer suggestions to protocol development and addressing areas for concern that arises in the implementation phase. As a public health professional and victim advocate, I will help to ensure patient-centeredness within the study approach and workflow in working with victims of intimate partner violence.

Sincerely,

Elizabeth Stern, MPH
Healthcare Program Coordinator
North Carolina Coalition Against Domestic Violence

MUSC

Medical University
Of South Carolina

Family Medicine
5 Charleston Center, Suite 263
MSC 192
Charleston, SC 29425-0192

Phone (843) 876-2912
http://www.musc.edu

Alyssa Rheingold, Ph.D.
National Crime Victims Research and Treatment Center
Medical University of South Carolina
67 President Street, MSC 861
Charleston, SC 29425

August 29, 2017

Dear Dr. Rheingold,

I am very happy to submit this letter of support for your AHRQ grant application, and to serve on the Advisory Board of this project, if funded. As you know, I serve as the current Chair of the Department of Family Medicine at the Medical University of South Carolina (MUSC). In my position, I have clinical expertise in working with victims of intimate partner violence and their families, as well as extensive experience in the administrative and technological aspects necessary to carry out this important project. I work with an electronic health records system (EHR) on a daily basis and am aware of the potential for utilizing this system as proposed in the current project.

I am excited to hear that you are proposing to develop and evaluate a systematic process for using an EHR in combination with validated assessment tools to screen patients and alert providers of potential problems and patient referrals. Your qualified team has expertise in developing such a systematic process, both in your extensive experience with victims of intimate partner violence and in your qualified and lengthy experience in informatics and EHR systems. I look forward to working with you and your team as a member of the Advisory Board.

I understand that the Advisory Board will meet on a quarterly basis, were we will discuss grant progress, identify safety concerns, and raise any potential barriers to successful program implementation. In addition, the Advisory Board will ensure patient-centeredness throughout program implementation and evaluation, as well as discuss feasibility and safety of the procedures for victims of intimate partner violence.

As a member of the Advisory Board, I will help ensure that all procedures and interventions are centered on the patient, and that they are both feasible and safe for victims of intimate partner violence. My expertise in working with this population will assist in successfully reaching these goals. I am committed to attending quarterly meetings to review progress and identify safety concerns, as well as to review any information pertinent to the project.

Given the team's impressive expertise, as well as project goals and objectives, this project is an ideal fit for this mechanism. There is no question you will successfully meet and exceed the aims of your project should our application be funded. I am excited to be a part of that process.

Sincerely,

Terrence E Steyer MD

Terrence Steyer, M.D.
Gregory – Chandler Endowed Chair in Family Medicine
Department of Family Medicine

MUSC Health
MEDICAL UNIVERSITY of SOUTH CAROLINA

Robert Warren MD, PhD, MPH
Chief Medical Information Officer
Professor of Pediatrics/Rheumatology
Professor of Public Health Science/Informatics
warrenrw@musc.edu

261 Calhoun Street
Suite 316
Charleston SC 29425
Tel 843 876 1985
Fax 843 876 1374

August 30, 2017

Alyssa Rheingold, Ph.D.
Medical University of South Carolina
National Crime Victims Research and Treatment Center
67 President Street – 2 South
Charleston, South Carolina 29425

Dear Drs. Lenert and Rheingold,

I would like to offer my enthusiastic support for your team's application for the AHRQ grant focused on utilizing electronic medical records to assist with evaluating and service provision for victims of intimate partner violence. As the Chief Medical Information Officer for the Medical University of South Carolina (MUSC), my focus is on optimal utilization of information technology to improve health care and advance medical education and research, as well as optimization of health services for children with chronic illness. As such, I have utilized the Epic electronic medical records system for assessing and evaluating outcomes within the health care setting on a daily basis and am fully aware of the significant impact of this work. This proposal uses well-established methods to assess and evaluate the measurement of intimate partner violence, which is a novel and unique topic area of critical importance to public health. I look forward to collaborating with this team, and serving as an Advisory Board member, on this important project.

As we have discussed, I will be serving as a member of the Advisory Board on the current project. In this capacity, I will advise the team during the initial planning period and throughout project duration via quarterly Advisory Board meetings. Specifically, I will actively participate in quarterly meetings and tasks in the interim to review project progress, identify safety concerns, assist in interpreting data, and make suggestions to help ensure patient-centeredness, feasibility, and safety of the procedures and intervention.

I am excited about the proposed project and am enthusiastic about the potential impact that this project will have on assessing and referring victims of intimate partner violence. I am especially excited about the opportunity to utilize Epic, our electronic medical record system, to identify, assess, and refer victims to much needed services. The system lends itself very well for development of this systematic process. I look forward to sharing my experience and knowledge as part of the Advisory Board, and I offer my full support of this application.

Sincerely,

Robert Warren, M.D. PhD, MPH
Chief Medical Information Officer
Professor of Pediatrics / Rheumatology
Professor of Public Health Sciences / Informatics
Medical University of South Carolina

Epic

September 14, 2017

Leslie Lenert MD, MS, FACP, FACMI
Chief Research Information Officer
Medical University of South Carolina

Alyssa Rheingold, PhD.
National Crime Victims Research and Treatment Center
Medical University of South Carolina

Re: R18 HS025654-01A

Dear Drs. Lenert and Rheingold:

On behalf of Epic, I am pleased to offer this Letter of Support for your R18 application entitled ***"EHR-based screening and intervention for intimate partner violence."*** The proposed study aims to design, implement and systematically evaluate a comprehensive approach to intimate partner violence (IPV) screening and intervention. The objectives are not only to improve detection of IPV cases by incorporating patient self-reported assessments, but also to increase referral of those cases to appropriate counseling services.

Epic is an electronic health record (EHR) developer located in Verona, Wisconsin. We estimate that, when fully rolled out, our customer community will provide care for 50-60% of the U.S. population. As the EHR vendor for the Medical University of South Carolina (MUSC) we enthusiastically support your efforts to improve effectiveness of screening and intervention for IPV. The EpicCare® EHR and MyChart® integrated patient portal provide a broad range of functionality that can support your aims of implementing a more comprehensive screening strategy as well as driving decision support and referral guidance for clinicians based on that screening.

You have assembled a strong team and advisory group, including Dr. Robert Warren, Chief Medical Information Officer at MUSC, who have extensive experience using the Epic platform to support this type of initiative. We would be pleased to provide expert advice and guidance at any time regarding the technical capabilities of our system as you pursue the aims of your study.

We encourage you to share the outcomes of your effort with the larger Epic community during the annual Epic User Group Meeting or an appropriate Epic Advisory Council. Should your screening and intervention strategy prove successful in improving intervention and outcomes, we would be happy to facilitate the dissemination of the details of your program through Epic's community sharing platform and, as appropriate, making technical components of your intervention available via our Foundation System.

We look forward to contributing to this important project.

Sincerely,

Nancy Smider

Nancy Smider, PhD
Director, Research Informatics
Epic

MUSC
SOUTH CAROLINA CLINICAL &
TRANSLATIONAL RESEARCH INSTITUTE
An NIH Clinical & Translational Science Award
(CTSA) Consortium Member

Kathleen T. Brady, MD, PhD
Distinguished University Professor

Vice President for Research

**Director, South Carolina Clinical &
Translational Research (SCTR) Institute**

Colcock Hall
179 Ashley Avenue, MSC 002
Charleston, SC 29425-0002
Tel 843 792-5205
Fax 843 792-4817
bradyk@musc.edu

August 3, 2017

Dr. Bryan B. Kim
Agency for Healthcare Research and Quality
5600 Fishers Lane
Rockville, MD 20857

Dear Dr. Kim:

As Principal Investigator of the South Carolina Clinical & Translational Research Institute (SCTR) and Vice President for Research at MUSC, I am pleased to express my most enthusiastic support for his application entitled, "**EHR-based screening and intervention for intimate partner violence.**" Dr. Lenert's leadership of the Biomedical Informatics Center, a critical university resource and a central component of the SCTR Institute, has ensured novel approaches to disseminating research findings and requisite open source products to accomplish this goal have been a tremendous asset to our campus, and across that state of South Carolina. This initiative is truly translational, founded in team science best practices and exactly the type of program we are seeking to implement at MUSC and across our region.

I will work to assure that the resources needed for this application will be readily available. I commend Dr. Lenert and his team on their vision and determination to catalyze solutions that bring about healthier living and an environment of fully-engaged and empowered community stakeholders. Best of luck with your application!

Sincerely,

Kathleen T. Brady, MD, PhD
Distinguished University Professor
Director, South Carolina Clinical & Translational Research Institute
Vice President for Research

Office of the Sheriff **County of Charleston**

Sheriff J. Al Cannon, Jr.

Alyssa Rheingold, Ph.D.
National Crime Victims Research and Treatment Center
Medical University of South Carolina
67 President Street, MSC 861
Charleston, SC 29425

29 August 2017

Dear Dr. Rheingold:

As a victim advocate at the Charleston County Sherriff's Victim Office, I am very excited to serve on the Advisory Board for this important project that will improve and expand services for victims of intimate partner violence. Victims of intimate partner violence endure tremendous physical and mental health consequences as a direct result of violence perpetrated against them. Often, these victims are untreated because of the lack of standardized assessments, a systematic process for screening patients in health care settings, or an effective process for referring victims into counseling.

As a Law Enforcement Victim Advocate (LEVA), at the Charleston County Sheriff's Office, I refer many of our crime victims to different agencies for treatment and follow-up care. I have worked in collaboration with organizations, including the National Crime Victims Research and Treatment Center (NCVC), over the past 17 years in organizing the current Survivors of Homicide Support Group (SHSG) and intensive case management services for victims of violence, including victims of partner violence. In collaboration with community organizations and the NCVC, we have provided support groups, hosted annual candlelight vigils, annual SHSG picnics, workshops at Victims Rights Week, and development of materials such as the *Guide for Survivors of Homicide* and the *Needs Assessment of Survivors of Homicide.* I have worked with community organizations to institutionalize intensive case management services for victims and their families residing in rural areas, which included providing direct services, developing protocols and providing trainings within our state and nationally. I am excited to work on this multidisciplinary Advisory Board to assist in providing further support and care for victims of intimate partner violence.

As a member of the Advisory Board, I will help ensure that all procedures and interventions are centered on the patient, and that they are both feasible and safe for victims of intimate partner violence. My expertise in working with this population will assist in successfully reaching these goals. I am committed to attending quarterly meetings to review progress and identify safety concerns, as well as to review any information pertinent to the project.

I look forward to being a part of this critical initiative to improve services for victims of intimate partner violence and am excited for the outcomes of this project.

Sincerely,

Easter LaRoche, CVAS
Victim Advocate Coordinator
Charleston County Sheriff's Office

Administrative Office
3691 Leeds Avenue
N. Charleston, SC 29405
~ Sheriff ~
Voice (843) 554-2230
Fax (843) 554-2243

Law Enforcement Division
3691 Leeds Avenue
N. Charleston, SC 29405
~ Patrol ~
Voice (843) 202-1700
Fax (843) 554-2234

Al Cannon Detention Center
3841 Leeds Avenue
N. Charleston, SC 29405
Voice (843) 529-7300
Fax (843) 529-7406

Judicial Center
100 Broad Street, Suite 381
Charleston, SC 29401
Voice (843) 958-2100
Fax (843) 958-2128

The National Domestic Violence
HOTLINE
1.800.799.SAFE (7233) • 1.800.787.3224 (TTY)

Leslie Lenert and
Alyssa Rheingold
67 President Street, MSC 861
Medical University of South Carolina
Charleston, SC 29425

September 1, 2017

Dear Dr. Lenert and Rheingold:

On behalf of the National Domestic Violence Hotline I am pleased to support your AHRQ proposal to examine best informatics practices to screening for intimate partner violence (IPV). We believe this proposal has significant national relevance to guide understanding of best practices for identification and intervention for IPV victims within primary care settings We are excited to be part of this study and look forward to collaborating with you and the research team assembled to conduct this project.

The National Domestic Violence Hotline took its first call in 1996 and within seven years took our one millionth call. Over the past twenty years has grown and expanded to be a national resource for local communities. We are the only 24/7 center in the nation that has access to service providers and shelters across the U.S. Our advocates who attend extensive training sessions are capable o providing the highest level of advocacy, crisis intervention, safety planning, and referral services for IPV victim callers. The Hotline is part of the largest nationwide network of programs and expert resources and regularly shares insight about IPV with government officials, law enforcement agencies, media, and the general public. We are a non-profit organization funded by federal grants, donations, and foundation support.

We are committed to participate in all phases of this study including collaborating with your research team. We are excited about the potential for understanding informatics practices in screening of IPV within healthcare settings given recommendations by the US Preventative Services Task Force in 2013. We understand that we will be involved a "warm hand-off" referral for those patients that screen positive for IPV within the family medicine practices of your study. During these calls will be provide advocacy, safety planning crisis intervention, and referral services as needed. We will collect aggregate data of calls made to us my participants in the study to provide. This data will not be tied to caller names or any identifying information in order to protect the confidentiality of the caller. Data collected will be kept separate from our standard database system. We will work closely as part of the research team to provide feedback on protocol and process of design as well as offer insight in interpretation of findings. We also will assist with dissemination of these important findings. We have been involved on a number of planning calls thus far and feel our partnership is strong to address the demands of such a project.

Again we are truly excited to partner with the Medical University of South Carolina on this significant and timely research project.

Sincerely,

Katie Ray-Jones
Chief Executive Officer
National Domestic Violence Hotline

P.O. Box 161810 | Austin, Texas 78716 | 512.453.8117 | thehotline.org
